# Supplementary material for: Ribozyme-mediated RNA synthesis and replication in a model Hadean microenvironment
Source: Nat Commun. 2023 Mar 17;14:1495. doi: 10.1038/s41467-023-37206-4 (PMC10023712; doi:10.1038/s41467-023-37206-4)

Supplementary information for

Ribozyme-mediated RNA synthesis and replication in a model Hadean microenvironment

Authors: Annalena Salditt[1], Leonie Karr[1], Elia Salibi[2], Kristian Le Vay[2], Dieter Braun[1*], and Hannes Mutschler[2*]

Affiliations:

[1]Systems Biophysics and Center for NanoScience (CeNS) Ludwig Maximilian University Munich, Geschwister-Scholl-Platz 1, 80539 Munich, Germany

[2]Department of Chemistry and Chemical Biology, TU Dortmund University, Otto-Hahn-Str. 4a, 44227 Dortmund, Germany

*Co-corresponding author. Email: dieter.braun@lmu.de, hannes.mutschler@tu-dortmund.de

Contents

1. Supplementary Figures 3
2. Supplementary Tables 17
3. Supplementary Methods 22
   3.1. Measurement of the bulk pH changes in AWIs 22
   3.2. Temperature simulations 22
   3.3. Supplementary Statistics and Reproducibility 23
4. Supplementary Notes 24
   4.1. Supplementary Note 1 24
   4.2. Supplementary Note 2 26
   4.3. Supplementary Note 3 27

Supplementary References 28

Appendix 29

List of Abbreviations:

RNA, ribonucleic acid; DNA, deoxyribonucleic acid; PAGE, polyacrylamide gel electrophoresis; nt, nucleotides; bp, base pairs; A, adenine; C, cytosine; G, guanine; U, uracil; DTT, ; NTP, ; Tm, temperature of melting; BHQ, Black Hole Quencher; Cy5, Cyanine-5; FAM, carboxy-fluorescein; TRIS, Tris(hydroxymethyl)aminomethan; TEMED, *N*,*N*,*N*′,*N*′-Tetramethylethylendiamin; TBE, TRIS-Borate-EDTA-Buffer; HH-min, minimal hammerhead ribozyme; HH-sub, substrate of the hammerhead ribozyme; AWI system, air-water interface non-equilibrium system;

1. Supplementary Figures

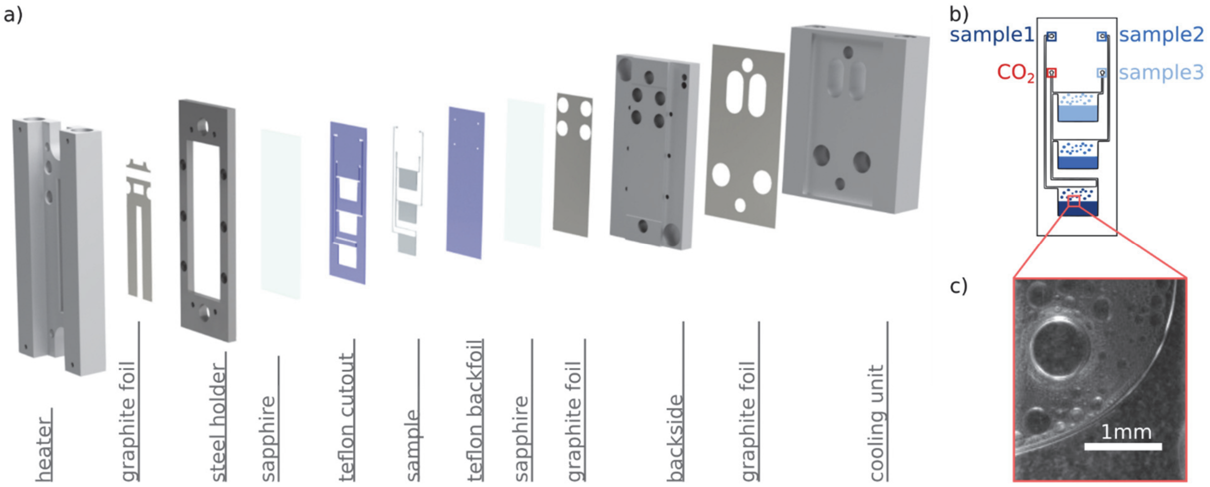

Supplementary Fig. 1: Construction of the microfluidic trap for the air-water interface (AWI) setup. a) The entire AWI setup was designed and implemented in Autodesk Inventor. The assembled trap consists of 12 parts as shown here, where the Teflon cutout and thus the sample shape can be adapted to the specific experimental needs. The AWI design was adapted from[1] **b)** The Teflon design allows to simultaneously screen three samples. All samples have a separate inflow and are connected to the same CO_2 inlet and are, thus, exposed to the same pressure conditions. **c)** The fog dynamics can be monitored with a microscope (fluorescence or bright field). In the compartments five phases can be distinguished: bulk phase, gaseous phase, interface, dew droplets that grow on the cold back side of the compartments and dew droplets that grow such that they touch both sides of the compartment. For more details, please refer to Method sub-section 'Preparation of AWI chambers and sample injection'.

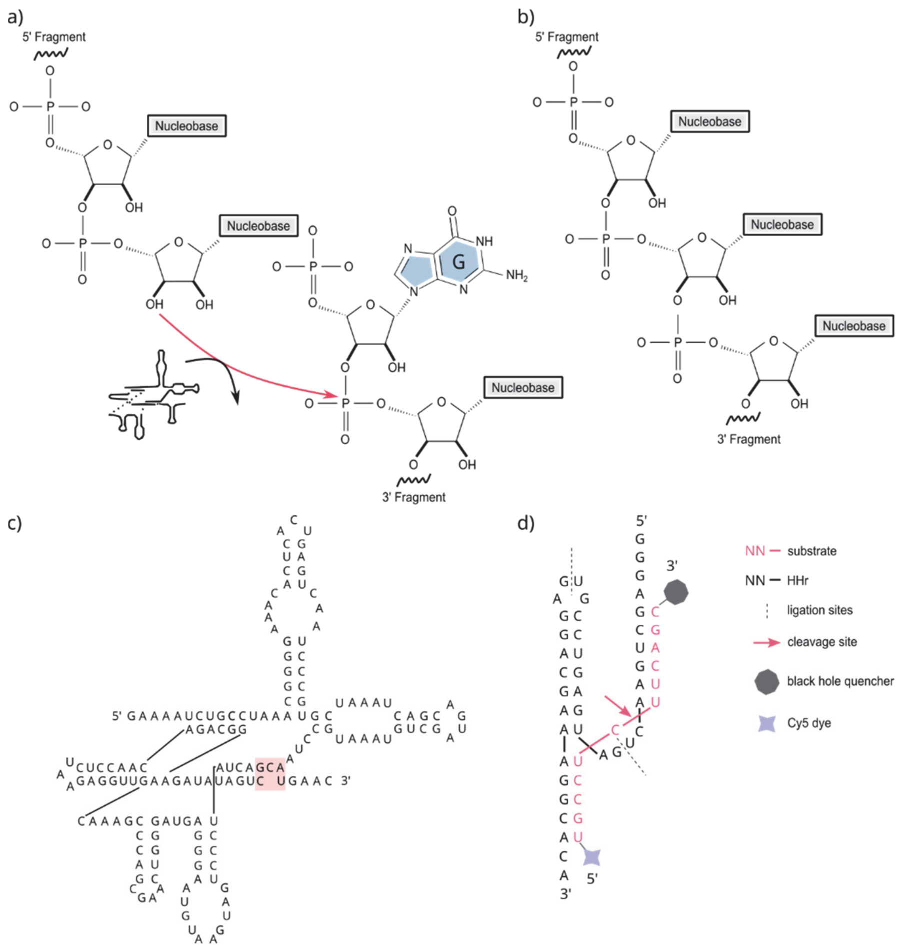

Supplementary Fig. 2: Detailed scheme of the reaction mechanism at the ligation site of the templated ligation catalyzed by the *sunY* ribozyme. a) After hybridization to the template (not shown), the 3' hydroxyl of the upstream fragment attacks the 3'end of the 5'end guanosine of the downstream substrate in the presence of *sunY*. **b)** After a transesterification reaction, where the 5' guanosine acts as a leaving group, both strands are covalently linked via a 3'-5' phosphodiester bond. The ligation efficiency can be significantly increased by introducing a G:U wobble pair at the 3' end of the attacking fragment. **c)** Secondary structure of the full-length *sunY* variant. The pink square highlights the G binding pocket that is essential for substrate-binding and positioning in the catalytic center. The sequence and secondary structure of the sunY were adapted from Doudna et al.[2]. **d)** Secondary structure of the hammerhead ribozyme variant (HH-min) used in this study. The HH-min cleavage substrate is shown in pink. The cleavage site is indicated by the pink arrow. The substrate has a 5' FAM- and 3' BHQ1-tag. The sequence and secondary structure of the minimal hammerhead ribozyme were adapted from Drobot et al.[3]. To ensure no residual function of the ligation substrates of the hammerhead ribozyme, the ligation sites were places such that they separate the active site of the hammerhead (dashed lines).

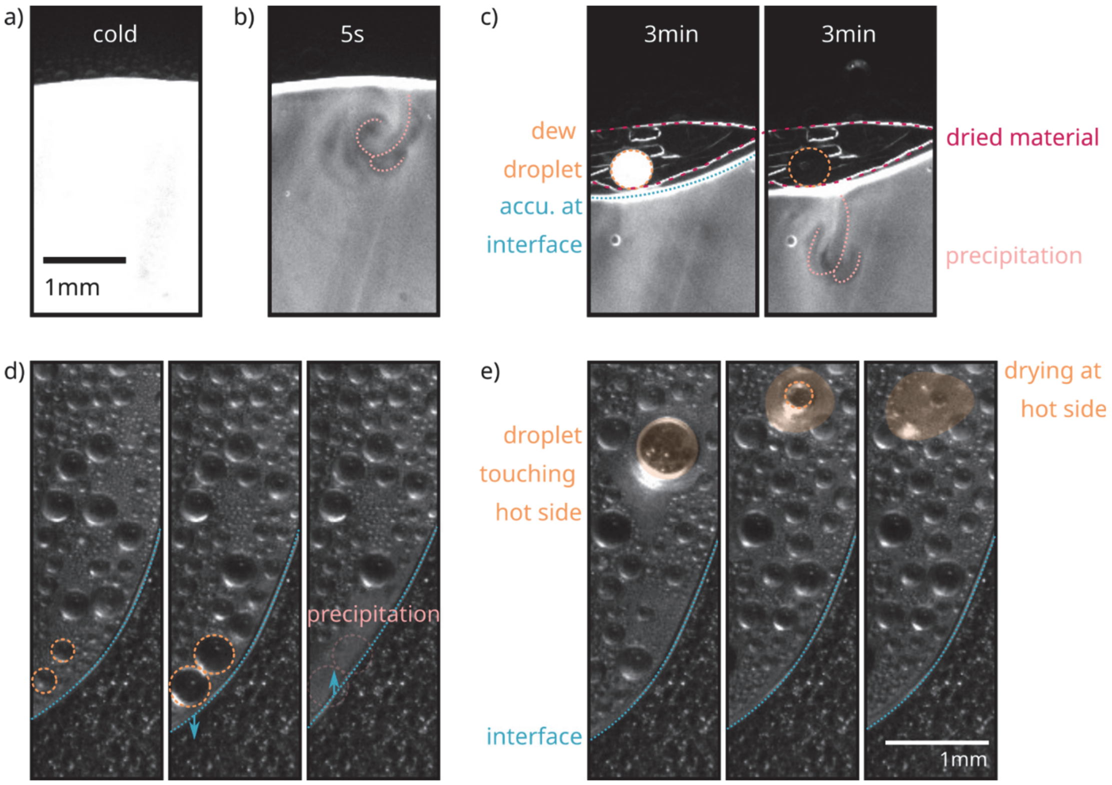

Supplementary Fig. 3: Visualization of the AWI dynamics was monitored either using the Cy5 channel (top) or in bright field (bottom). a) The interface of the semi-filled AWI was monitored using the Cy5-channel. Before heating, the bulk fluorescence shows a uniform distribution of RNA. **b)** Already after a few seconds, dew droplets form by condensation and precipitate back into the bulk solvent (precipitation). Simultaneously, RNA molecules start accumulate at the interface (blue line). The drop in total fluorescence is due to the temperature dependence of Cy5 fluorescence. **c)** As the interface moves up and down, some RNA dries on the hot sapphire window (pink line). Some dewdrops become so large that they touch the opposite side of the AWI chamber (left). If the droplet is close enough to the interface, the droplet resolves the dried material, and precipitates back into the bulk solution carrying the material with it. **d)** The dew droplets on the cold side can be visualized in bright field. They can grow, merge and again precipitate back into the bulk solution. The interface moves down as more water is evaporated and contained in the dew droplets and moves back up again as droplets precipitate. **e)** If a droplet touches both opposite sides of the chamber, it can also split. In this case, the droplet that forms at the front (hot) side of the chamber slowly dries due to evaporation. For more details, please refer to section 4.3. Source data are provided as a Source Data file.

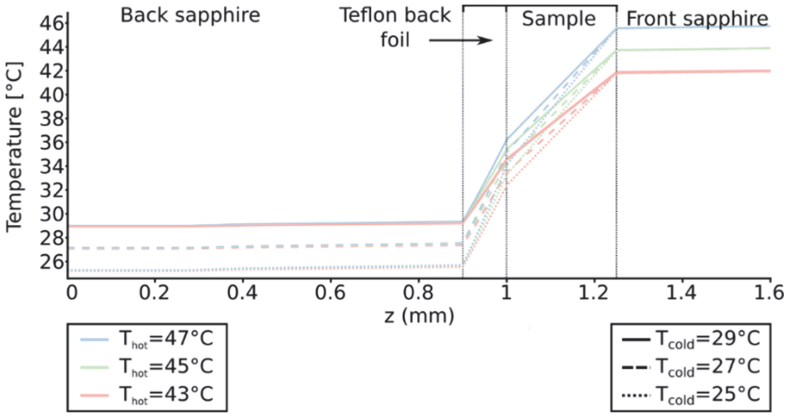

Supplementary Fig. 4: Simulation of temperature gradient inside the non-equilibrium chambers. Temperatures were simulated with finite-element simulations for different sets of experimentally measured temperature at the cold back and hot front side of the sapphire, respectively. The temperature gradient influences the accumulation behavior as well as the dew droplet dynamics. The gradient was optimized experimentally to achieve good droplet dynamics, i.e. large enough condensation droplets for the rearrangement to the hot side. Good overall droplet dynamics were found for temperatures between 45-47 °C and 26-28 °C at the front and back sapphire windows, respectively. For more details, please refer to section 3.2. Source data are provided as a Source Data file.

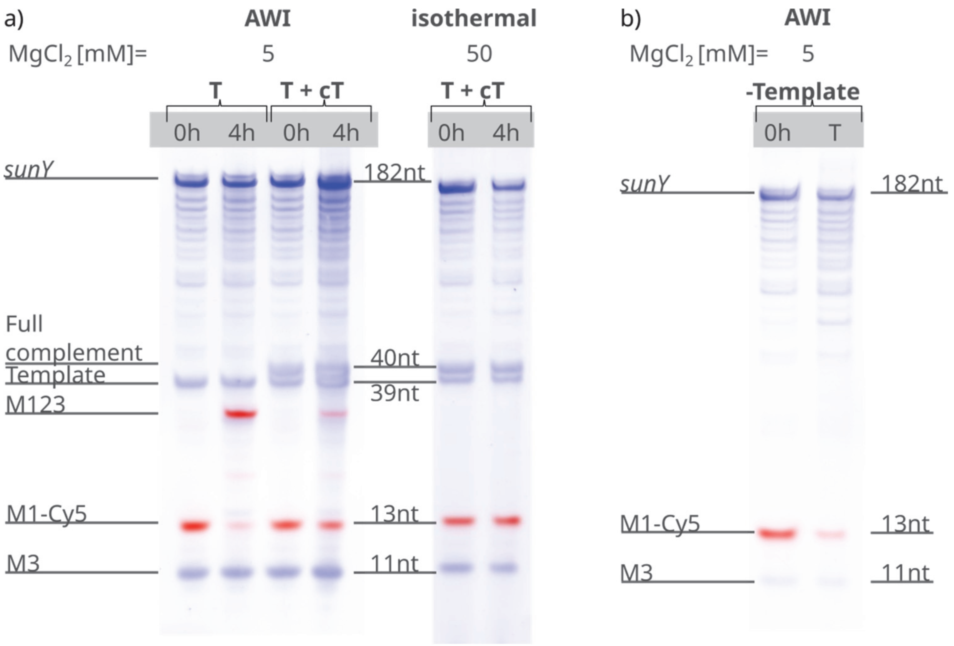

Supplementary Fig. 5: Characterisation of *sunY* catalysed RNA ligation using the model ligation reaction of M123. a) The maximum temperature in the reaction setup was in both cases (isothermal and AWI) well below the calculated melting temperature (76 °C) of the M123-template duplex. The ligation reaction was investigated starting from three ligation substrates (M1-3). For quantification, the first substrate was labeled with a 5' Cy5 tag. The ligation substrate concentrations were each at 20 µM, with a template concentration of 5 µM. For the strand separation control, the concentration of the full complement was also 5 µM. The reaction under AWI conditions proceeded both, in absence (left) (n=2) and presence (right) of reverse complement of the template (n=2), although with lower efficiency in the case of the reverse complement being present. The yield for the full-length product (M123) was 82% and 30% with and without the complement present, respectively. This suggests that the dew droplet dynamics are sufficient to provide enough strand separation capacity to allow for the synthesis and multiple turnover. The isothermal control shows neither product nor intermediate formation (n=2). **b)** The negative (no template) control for the simple ligation system shows no ligation product (n=1). This confirms that even with strong accumulation inside the non-equilibrium compartment, the ligation still proceeds in a templated manner and therefore ensures information transfer instead of just an elongation behavior. Source data are provided as a Source Data file.

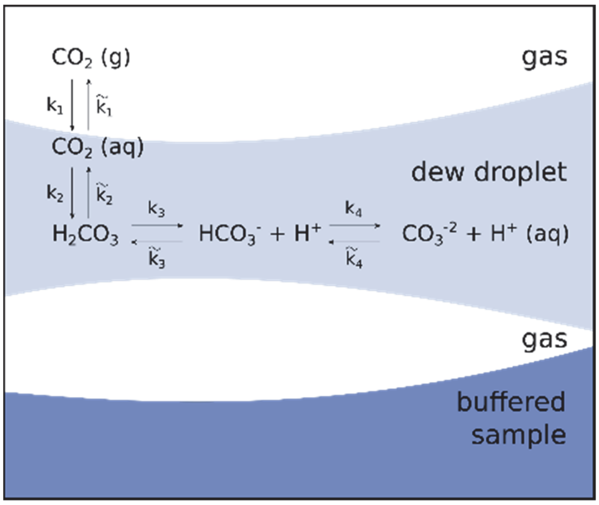

Supplementary Fig. 6: Schematic representation of the reactions leading to acidification of the dew droplets in the presence of CO_2. The dissolution of gaseous carbon dioxide into dew leads to a reduction of the pH of the dew droplets depending on the applied pressure. Reaction scheme and pH dependence of the dew droplets were adapted from[4]. For more details, please refer to section 3.1.

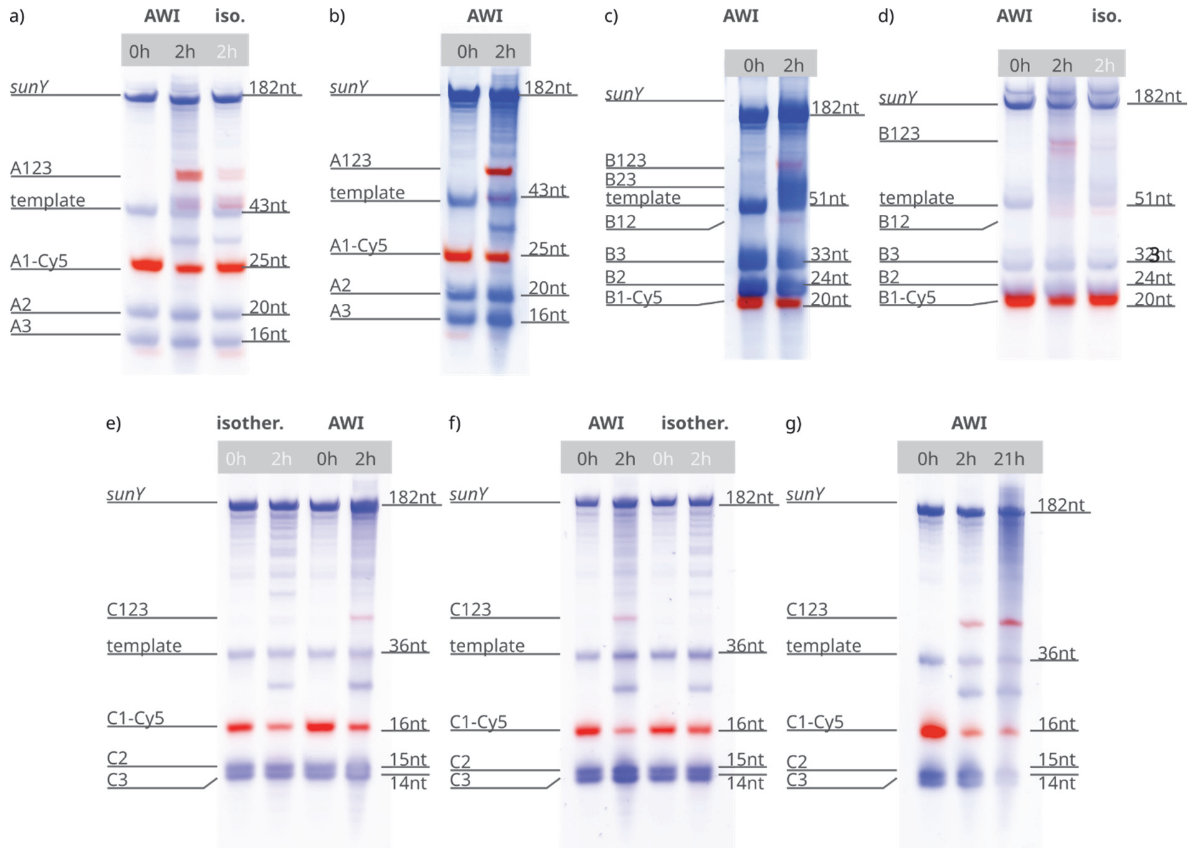

Supplementary Fig. 7: Synthesis of individual *sunY* fragments. a) *sunY* catalyzed ligation of *sunY* fragment A123. Yields under AWI-conditions were 37%, compared to 8% under isothermal conditions. **b)** Replicates of A123 synthesis under AWI conditions. Here, the yield was 43%. **c)** Synthesis of fragment B123 in the AWI system shows full length product with a yield of 17%. **d)** Synthesis of B123 under AWI and isothermal conditions showed yields of 22% and 1%, respectively. **e)** Duplicates of the reaction under AWI conditions and isothermal conditions of fragment C123. The yield of the reaction under AWI conditions was 12%, the isothermal control showed a yield of 2%. **f)** Triplicates of the reaction from e) performed under AWI and isothermal conditions. Yields were 20% under AWI conditions and 0.6% under isothermal conditions. **g)** Comparison of 2 h reaction time to 21 h reaction time. For the 21 h hour reaction, the CO_2 supply was stopped overnight. The yields were 27% and 52%, respectively. Comparing the synthesis in isothermal conditions of C123 and B123 to A123 shows that the synthesis of A is most effective probably due to the less efficient inhibitory effect of the template resulting from differences in sequence of the section corresponding to A of the full-length *sunY* and the fragmented *sunY* version used in the experiments. Source data are provided as a Source Data file.

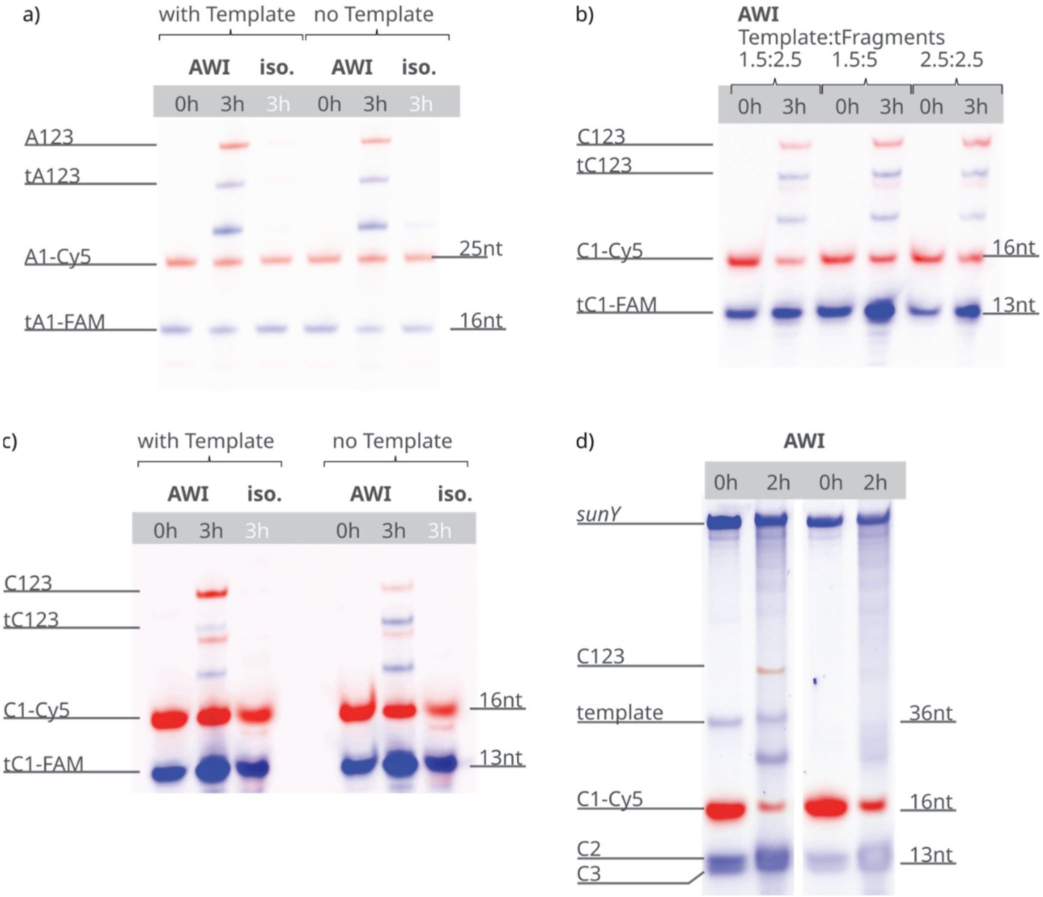

Supplementary Fig. 8: Synthesis of *sunY* fragments A123, and C123, and the respective reverse complements. **a)** For a full replication cycle, we included 10 µM ligation substrates for both the sense and antisense template strands. The concentrations of templates and *sunY* were 2.5 µM. In the AWI system, synthesis of the *sunY* fragment A123 and the reverse complement template tA123 proceeded with and without seeding of initial tA123. With seeding, the yields in the AWI system were 40% and 19% for full-length A123 and tA123, respectively. Under isothermal conditions, no full-length product tA123 was detected. The yield for A123 under isothermal conditions was 6%. Without template, the yields in the AWI system were 34% and 24% for A123 and tA123, respectively. The isothermal control showed no full-length product for both A123 and tA123. **b)** For the synthesis of *sunY* fragment C123 and its complement tC123 at 10 µM substrates did not lead to any ligation products. Thus, multiple concentrations of template substrates tC1-3 were tested in order to decrease *sunY* inhibition by the partially complementary RNA strands. The cumulative concentration of all complementary strands was always kept above 2.5 µM. The respective concentration of the substrates C1-3 and *sunY* were kept at 10 µM and 2.5 µM, respectively. All reactions showed full-length product with yields for C123 of 26%, 27% and 34% and tC123 of 7%, 4% and 8% (from left to right). **c)** C123 synthesis using 2.5 µM template and *sunY*, 5 µM tC1-3 substrates and 10 µM of C1-3 substrates. The yields for C123 in the AWI system were 11% and 3% with and without template. The yields for tC123 in the AWI system were 1% and 3% with and without template. Under isothermal conditions, no full-length products were detected. **d)** To exclude that the ligation in the AWI proceeds without template (e.g. just through the concentration effect in the AWI system), a no-template control was performed for fragment C123. However, ligation was observed in presence of template tC123 only (left two lanes). No ligation was detected without tC123 present (right two lanes). Source data are provided as a Source Data file.

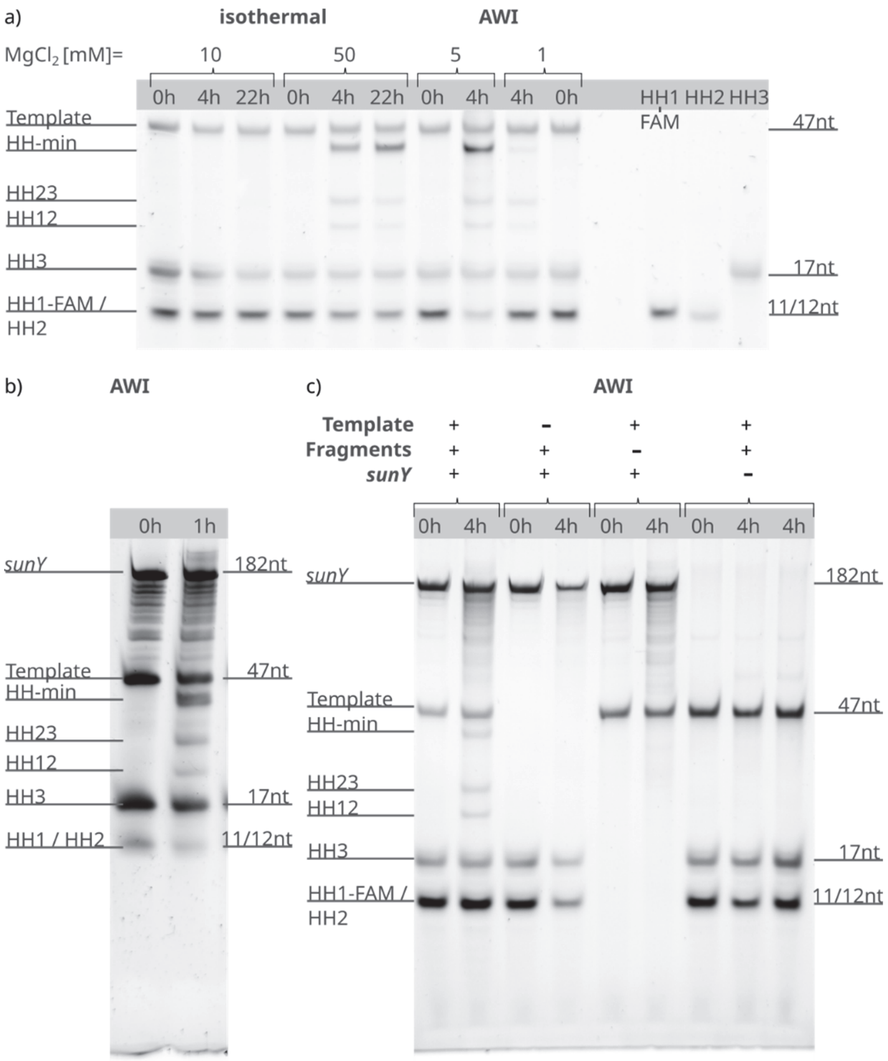

Supplementary Fig. 9: Ligation of HH-min by *sunY*. Reactions with HH-min template (5 µM) and the three HH-min (HH1-3) substrates (10 µM, 5'-FAM-HH1) were carried out in 30 mM Tris·HCl pH 7.5 and 100 mM KCl at different $MgCl_2$ concentrations as indicated. The temperature under isothermal conditions was 45 °C. A temperature gradient of 28 °C and 46 °C between the cold and warm outer side of the sapphires was chosen for the AWI system. The CO_2 pressure was adjusted to 0.5-0.6 bar above atmospheric pressure. To exclude potential dye artifacts, we ran the HH-min synthesis with a 5' FAM label at the first substrate HH1 (**a), c)**) or without any label (**b)**). In both cases, the reaction yielded full-length product (HH-min). **a)** Under isothermal conditions, the reaction was carried out in the presence of 50 mM $MgCl_2$. Under non-equilibrium conditions, the reaction produces significant HH-min full-length product at 5 mM $MgCl_2$ and even at lower magnesium concentrations of 1 mM, whereas no product was observed under isothermal conditions. **b)** Synthesis using unlabeled HH1 (5 mM $MgCl_2$). **c)** To ensure that the accumulation in the non-equilibrium did not lead to any unintended side reactions, each RNA component was omitted individually. Removing either template, substrates or *sunY* completely abolishes ligation (1 mM $MgCl_2$.). Source data are provided as a Source Data file.

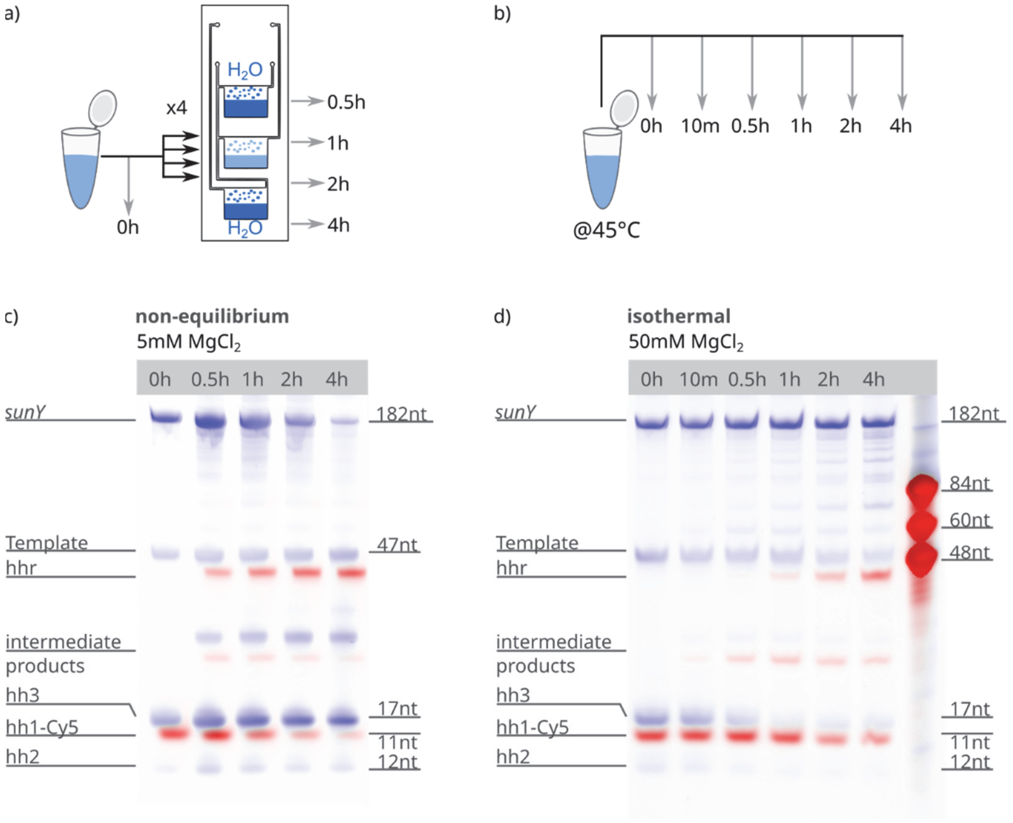

Supplementary Fig. 10: Kinetics of HH-min synthesis with a 1:2 ratio of template to substrates. To explore the kinetic differences of HH-min synthesis in isothermal vs. non-equilibrium conditions, samples were analyzed at different time points of the reaction. Here, the ratio of substrates to template was 1:2 with concentrations of 10 µM for the HH-min ligation substrates and 5 µM for the template. The isothermal reaction was prepared in 30 mM Tris·HCl pH 7.5, 100 mM KCl and 50 mM MgCl2 and incubated at 45 °C. **a)** For reactions performed in the AWI-system, samples were aliquoted into equal parts for the time points 30 min, 1 h, 2 h and 4 h. For each time point a separate AWI-system was assembled. To ensure similar humidity conditions for all experiments, empty chambers were filled with 15 µL of water. No samples from incubation times shorter than 0.5 h were taken as the heating and cooling for the temperature gradient at the beginning and end of the experiment introduces delays, which obscured results from short incubation periods. **b)** For reactions carried out under isothermal condition, time points were taken from the same reaction at 0 h, 10 min, 0.5 h, 1 h, 2 h, and 4 h. **c)** PAGE analysis of samples carried out under AWI-conditions. **d)** PAGE from samples from isothermal incubations. Source data are provided as a Source Data file.

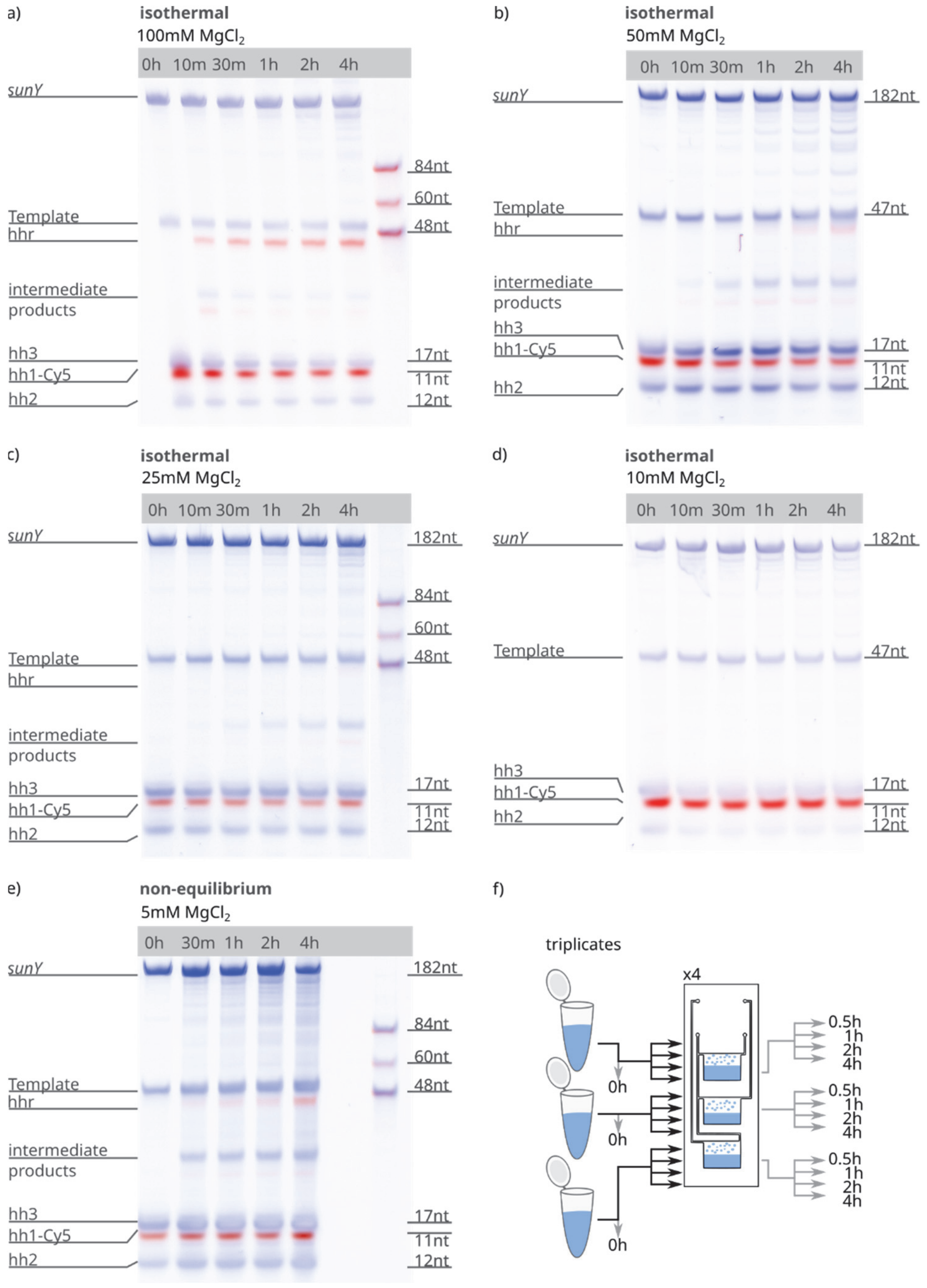

Supplementary Fig. 11: Kinetics of the HH-min synthesis with a 1:4 ratio of template to substrates. To explore the kinetic differences of HH-min synthesis in isothermal vs. non-equilibrium conditions, samples were analyzed at different time points of the reaction. Here, the ratio of ligation substrates to template was 1:4 with concentrations of 12 µM for the HH-min ligation substrates and 3 µM for the template. Samples from reactions carried out under isothemal conditions were analyzed by PAGE for buffers containing **a)** 100 mM $MgCl_2$, **b)** 50 mM $MgCl_2$, **c)** 25 mM $MgCl_2$ and **d)** 10 mM $MgCl_2$. **e)** PAGE analysis of HH-min synthesis carried out in presence of 5 mM $MgCl_2$ **f)** Illustration of the AWI setup that allows to run parallel triplicates under non-equilibrium conditions. Triplicates of the reactions were prepared individually and divided into equal parts for the time points 30 min, 1 h, 2 h and 4 h. For each time point a separate AWI was prepared with the three chambers corresponding to the three triplicates. Source data are provided as a Source Data file.

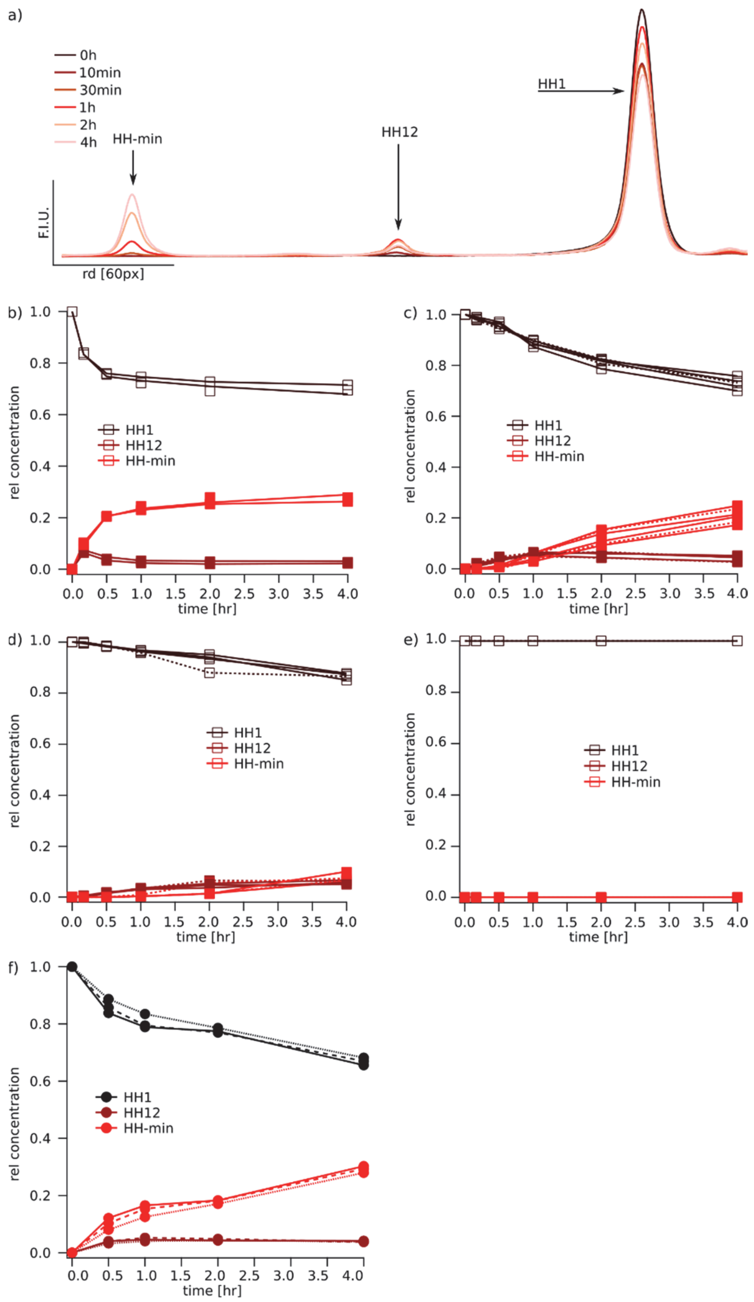

Supplementary Fig. 12: Quantification of HH-min product formation by PAGE for both isothermal and AWI conditions. a) Example traces of the PAGE analysis for one time series of an isothermal reaction carried out in presence of 50 mM $MgCl_2$. Each peak was fitted with one, or in the case of broader shoulders, several Gaussians. The area corresponding to one peak was normalized with the total sum of all gaussians. **b)** Results for 100 mM $MgCl_2$ show an average yield of 27 ± 1% after 4 h. **c)** Results for 50 mM $MgCl_2$ with an average yield of 20 ± 3% after 4 h. **d)** Results for 25 mM $MgCl_2$ with an average yield of 8 ± 2% after 4 h. **e)** Results for 10 mM $MgCl_2$ with 0% yield for all time points. **f)** Triplicates for the AWI reaction with an average yield of 29 ± 1% after 4 h. Source data are provided as a Source Data file.

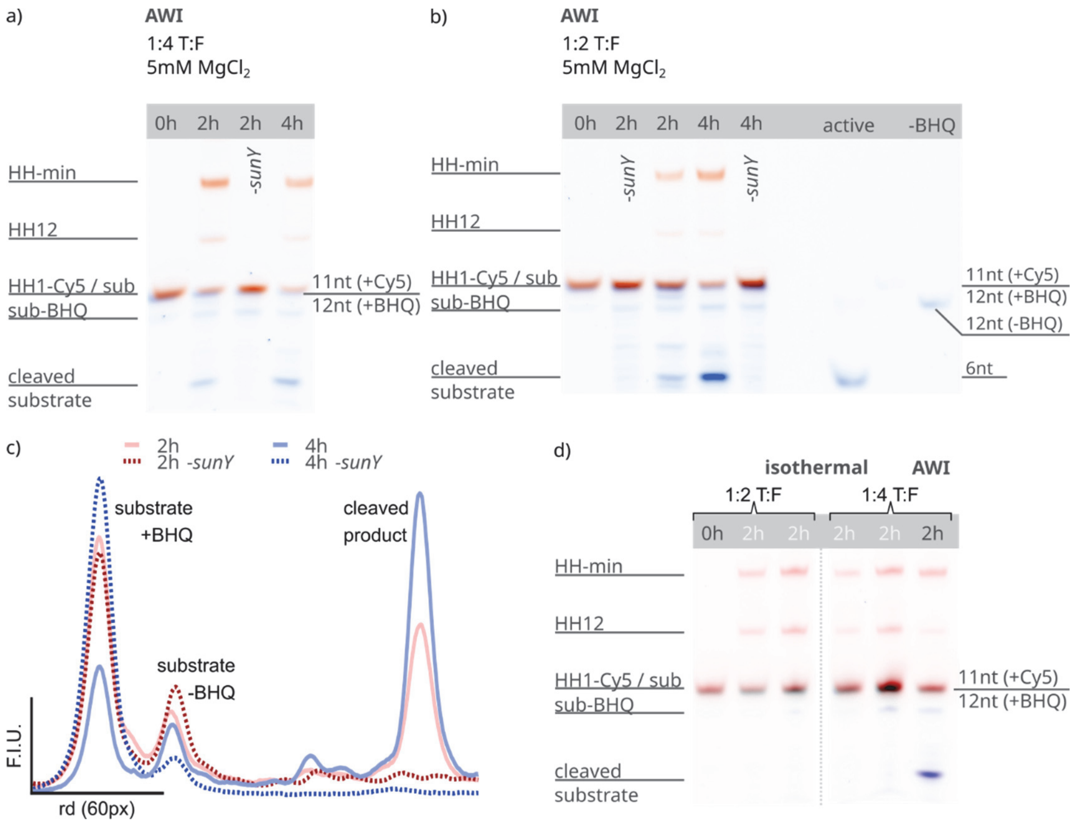

Supplementary Fig. 13: One-pot HH-min synthesis and RNA cleavage under different conditions. PAGE analysis of reaction in AWI chambers shows HH-min synthesis (5'-Cy5 labeled, red) as well as the HH-sub cleavage (5'-FAM labeled, 3'BHQ labelled, blue) for **a)** a 4-fold excess and **b)** a 2-fold excess of HH-min ligation substrates over template. Reaction times were either 2 h or 4 h. In absence of *sunY*, no HH-min or cleaved substrate was detected. A second band, running just below the HH-sub was confirmed to be the substrate devoid of BHQ, which partially dissociates during the reaction. Several replicates (i.e., 10 for 2 h running time and 4 for 4 h running time) were performed for the case of 2-fold excess of fragment to template. The resulting yields of the cleaved product as well as mean and standard deviation are listed in Supplementary Table 7. The identity of the cleaved substrate was confirmed by a separate isothermal control reaction using pre-synthesized full-length HH (biomers) and HH-sub carried out at 45 °C for 30 min. **c)** For quantification of the cleaved substrate, the extracted traces were fitted with Gaussians. The area corresponding to the first peak was multiplied with an experimentally determined correction factor of 1.69 to account for the quenched FAM fluorescence of uncleaved substrate. The yield is then calculated as the fraction of the area of the cleaved product divided by the total area (including bands due to hydrolysis). **d)** PAGE showing hammerhead synthesis and HH-sub cleavage under isothermal and non-equilibrium (AWI) conditions. Source data are provided as a Source Data file.

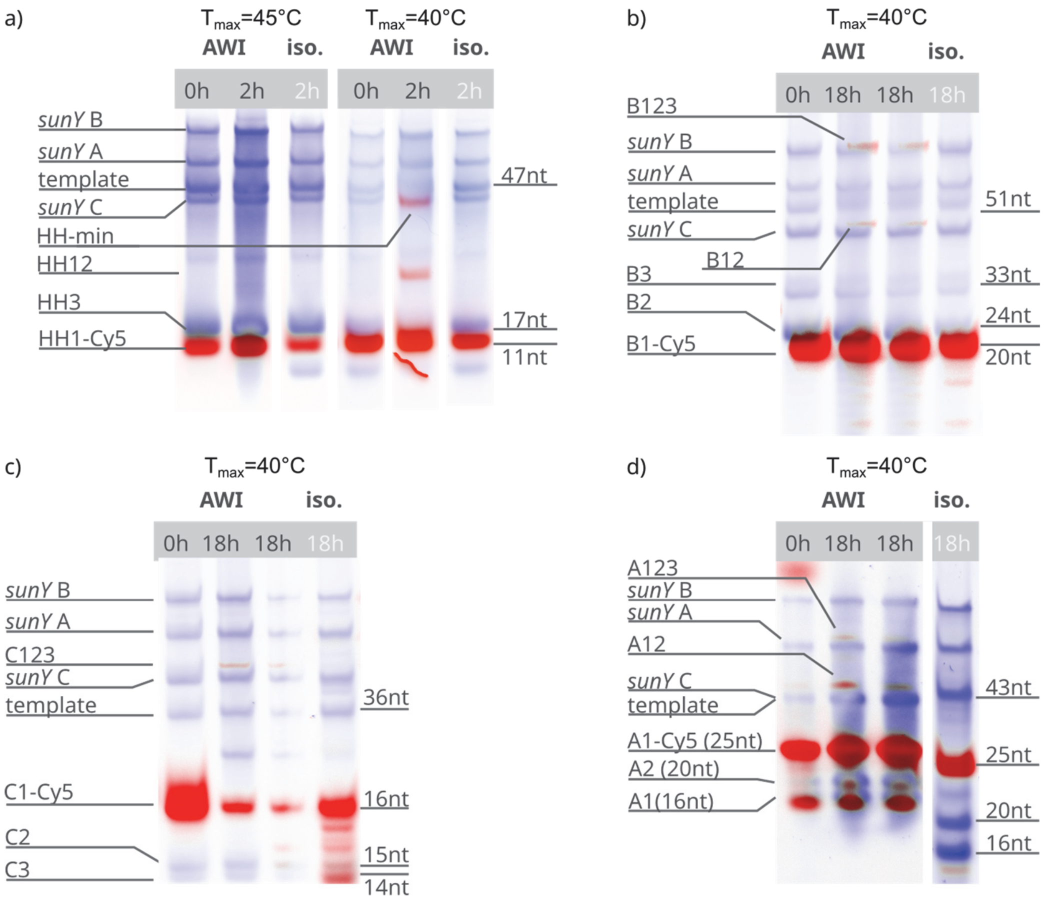

Supplementary Fig. 14: HH-min and *sunY* fragment ligation by the split *sunY* variant. a) Ligation of HH-min by the split (tripartite) *sunY* variant. No synthesis was observed for experimental conditions compatible with the full-length ribozyme (T_{max} = 45 °C). Reducing the temperature of the hot side of the AWI system to 40 °C led to the formation of full-length HH-min. The yield (7%) was significantly lower compared to the full-length version under standard conditions (~40%). No product was detected for isothermal conditions. **b)** Ligation of fragment B123, **c)** C123, and **d)** A123 using the split *sunY* system. The reaction time was 18 h. The CO_2 supply to the system was stopped overnight (reducing the strand separating capacity of the AWI system). For all *sunY* fragments traces of the full-length products were detected in the AWI system. No full-length products were detected under isothermal conditions. Source data are provided as a Source Data file.

2. Supplementary Tables

Supplementary Table 1: List of all RNA sequences used in the experiments. The second and third ligation substrates were ordered with an additional G base at the 5'-end (lowercase), which act as a leaving group during *sunY* catalyzed RNA-ligation.

Name	nt	Sequence (5'-3')
**A Template**	43	GAUUUUGCCCUUUGGGAGUGACUCUGUUAGGCAGCGAUUUAGU
**A1 (5'-Cy5)**	25	GAAAAUCUGCCUAAAACGGGAAACU
**A2**	20	gCUCACUGAGACAAUCCGUU
**A3**	16	gGCUAAAUCAGCAGUA
**tA123**	43	UGAUUUAGCGACGGAUUGUCUCAGUGAGGGUUUCCCGUUUUAG
**tA1 (5'-FAM)**	16	UGAUUUAGCGACGGAU
**tA2**	16	gUGUCUCAGUGAGGGU
**tA3**	13	gUUCCCGUUUUAG
**B Template**	51	ACGGGUUGCUGGUAGGGACUACUUACAUUCCCUCGUCCCAGUUCGCUGGGC
**B1 (5'-Cy5)**	20	GCUGUAAAUGCCCAACGACU
**B2**	24	gAUCCCUGAUGAAUGUAAGGGAGU
**B3**	33	gAGGGUCAAGCGACCCGAAACGGCAGACAACUC
**tB123**	52	UCGGGUCGCUUGACCCUGCUCCCUUACAUUCAUCAGGGAUGGUCGUUGGGCA
**tB1 (5'-FAM)**	20	UCGGGUCGCUUGACCCUGCU
**tB2**	20	gCCCUUACAUUCAUCAGGGA
**tB3**	14	gUGGUCGUUGGGCA
**C Template**	36	AACUUCUAUAUCGGACUUGACGUACCGCUGUACGUC
**C1 (5'-Cy5)**	16	GAGUUGAAGAUAUAGU
**C2**	15	gCUGAACUGCAUGGU
**C3**	14	gGACAUGCAGGAUC
**tC1 (5'-FAM)**	13	CUGCAUGUCGCCA
**tC2**	14	gUGCAGUUCAGGCU
**tC3**	11	gAUAUCUUCAA
**tC123**	36	CUGCAUGUCGCCAUGCAGUUCAGGCUAUAUCUUCAA
**HH-sub (5'FAM and 3'BHQ)**	12	UGCCUCUUCAGC
**HH-min**	38	GGAGCUGAACUGAUGAGUCCGUGAGGACGAAAGGCACA
**HH Template**	47	GGGAAAUGUGCCUUUCGUCCUCGCGGACUCAUCGGUUCAGCUCCAAA
**HH1 (5'-Cy5)**	11	GGAGCUGAACU
**HH2**	12	gGAUGAGUCCGU
**HH3**	17	gGAGGACGAAAGGCACA
**M123**	40	GCGCGCGAAGGAAGGAUUGGUAUGUGGUAUAUUCGCGCGC
**M Template**	39	GGGAAACGAAUAUACCGCAUACCGAUCCUUCCUUCGAAA
**M1 (5'-Cy5)**	13	CGAAGGAAGGAUU
**M2**	8	gGGUAUGU
**M3**	11	gGGUAUAUUCG
sunY	182	GGG AAA AUC UGC CUA AAC GGG GAA ACA CUC ACU GAG UCA AUC CCG UGC UAA AUC AGC AGU AGC UGU AAA UGC CUA ACG ACU AUC CCU GAU GAA UGU AAG GGA GUA GGG UCA AGC GAC CCG AAA CGG CAG ACA ACU CUA AGA GUU GAA GAU AUA GUC UGA ACU GCA UGG UGA CAU GCA GGA UC

Supplementary Table 2: Melting temperature predictions for the *sunY* ligation substrates A1/2/3, B1/2/3, and C1/2/3 with their respective templates. Calculations were performed for pH 7, pH 4 and pH 3.6, a monovalent ion concentration of 100 mM, adapted length and GC content and varying divalent ion concentration. The last columns correspond to melting temperatures for pH 4 and pH 3.6 with low concentrations of monovalent (1 mM) and divalent (0.2 mM) metal ions. If no melting transition was observed, the melting temperature is reported as NaN. All reported values are for the longest possible double stranded region (32 bp; 13 bp) and assume stoichiometric conditions.

**template present**	**A123**	**A1**	**A2**	**A3**	**length [nt]**	**GC-content**	**c_{MgCl2} [mM]**	**$T_{M,pH7}$ [°C]**	**$T_{M,pH4}$ [°C]**	**$T_{M,pH3.6}$ [°C]**	**$T_{M,pH4,\ low\ salt}$ [°C]**	**$T_{M,pH3.6,\ low\ salt}$ [°C]**
yes	yes				57	43%	50	85.72	57.98	29.64	42.61	15.53
							10	84.53	56.88	28.63		
							5	83.56	55.99	27.81		
							1	79.23	51.97	24.13		
yes		yes			25	40%	50	72.98	45.10	17.37	30.06	3.51
							10	71.83	44.04	16.39		
							5	70.89	43.17	15.60		
							1	66.68	39.25	12.00		
yes			yes		19	47%	50	69.89	41.57	13.72	26.47	NaN
							10	68.74	40.51	12.75		
							5	67.81	39.64	11.95		
							1	63.61	35.73	8.35		
yes				yes	15	40%	50	53.10	25.13	NaN	10.48	NaN
							10	52.00	24.12	NaN		
							5	51.11	23.28	NaN		
							1	47.08	19.51	NaN		
**template present**	**B123**	**B1**	**B2**	**B3**	**length [nt]**	**GC-content**	**c_{MgCl2} [mM]**	**$T_{M,pH7}$ [°C]**	**$T_{M,pH4}$[°C]**	**$T_{M,pH3.6}$ [°C]**	**$T_{M,pH4,\ low\ salt}$ [°C]**	**$T_{M,pH3.6,\ low\ salt}$ [°C]**
yes	yes				75	52%	50	95.03	66.34	37.35	50.61	22.93
							10	93.81	65.21	36.32		
							5	92.81	64.29	35.48		
							1	88.37	60.18	31.71		
yes		yes			20	50%	50	73.72	45.24	17.16	30.01	3.10
							10	72.56	44.16	16.17		
							5	71.62	43.29	15.37		
							1	67.38	39.34	11.74		
yes			yes		23	43%	50	73.06	45.01	17.18	29.91	3.26
							10	71.91	43.94	16.20		
							5	70.97	43.07	15.40		
							1	66.75	39.15	11.79		
yes				yes	32	59%	50	90.56	61.62	32.66	45.91	18.21
							10	89.35	60.50	31.64		
							5	88.36	59.59	30.80		
							1	83.95	55.49	27.04		
**template present**	**C123**	**C1**	**C2**	**C3**	**length [nt]**	**GC-content**	**c_{MgCl2} [mM]**	**$T_{M,pH7}$ [°C]**	**$T_{M,pH4}$[°C]**	**$T_{M,pH3.6}$ [°C]**	**$T_{M,pH4,\ low\ salt}$ [°C]**	**$T_{M,pH3.6,\ low\ salt}$ [°C]**
yes	yes				43	44%	50	83.88	55.77	27.51	40.44	13.43
							10	82.69	54.68	26.51		
							5	81.73	53.78	25.69		
							1	77.41	49.78	22.02		
yes		yes			16	31%	50	51.66	24.21	NaN	9.74	NaN
							10	50.57	23.20	NaN		
							5	49.69	22.38	NaN		

							1	45.69	18.64	NaN		
yes			yes		14	50%	50	53.71	25.15	NaN	10.33	NaN
							10	52.61	24.13	NaN		
							5	51.71	23.30	NaN		
							1	47.65	19.49	NaN		
yes				yes	13	54%	50	48.10	19.32	NaN	4.55	NaN
							10	47.01	18.31	NaN		
							5	46.12	17.48	NaN		
							1	42.11	13.70	NaN		

Supplementary Table 3: HH-sub cleavage yields from independent replicates of combined one-pot HH-min synthesis / cleavage at a stoichiometry of 1:2 template to ligation substrates.

	2h – yield cleaved product	4h – yield cleaved product
Exp 1	0.31	0.57
Exp 2	0.34	0.50
Exp 3	0.37	0.64
Exp 4	0.39	
Exp 5	0.60	
Exp 6	0.61	
Exp 7	0.28	
Exp 8	0.49	
Exp 9	0.46	
Exp 10	0.19	0.68
**mean**	**0.40**	**0.60**
**standard deviation**	**0.13**	**0.07**

Supplementary Table 4: Melting temperature predictions for the HH-min ligation system. Calculations were performed for pH 7, pH 4 and pH 3.6, a monovalent ion concentration of 100 mM, adapted length and GC content and varying divalent ion concentration. The last columns correspond to melting temperatures for pH 4 and pH 3.6 with low concentrations of monovalent (1 mM) and divalent (0.2 mM) metal ions. If no melting transition could be determined, the melting temperature is reported as NaN. All reported values are for the longest possible double stranded region (32 bp; 13 bp) and assume stoichiometric conditions.

**template present**	**HH-min**	**HH1**	**HH2**	**HH3**	**length [nt]**	**GC-content**	**c_{MgCl2} [mM]**	**$T_{M,pH7}$ [°C]**	**$T_{M,pH4}$ [°C]**	**$T_{M,pH3.6}$ [°C]**	**$T_{M,pH4,}$ low salt [°C]**	**$T_{M,pH3.6,}$ low salt [°C]**
yes	yes				38	55%	50	90.11	61.40	32.58	45.77	18.22
							10	88.91	60.28	31.56		
							5	87.93	59.38	30.73		
							1	83.52	55.30	26.98		
yes		yes			11	55%	50	14.91	NaN	NaN	NaN	NaN
							10	13.92	NaN	NaN		
							5	13.11	NaN	NaN		
							1	9.41	NaN	NaN		
yes			yes		11	55%	50	14.91	NaN	NaN	NaN	NaN
							10	13.92	NaN	NaN		
							5	13.11	NaN	NaN		
							1	9.41	NaN	NaN		
yes				yes	16	56%	50	67.67	38.17	10.16	NaN	NaN
							10	66.53	37.11	9.19		
							5	65.60	36.25	8.39		
							1	61.40	32.33	4.78		

3. Supplementary Methods

3.1. Measurement of the bulk pH changes in AWIs

The pH of the dew droplets acidifies in the presence of CO_2 as the gaseous CO_2 dissolves in water. The pH drop depends on the pressure of CO_2 inside the chamber. In our case, the applied CO_2 pressure is 0.5-0.7 bar, leading to mean pH values between 4 and 4.5 [4].

If the buffer capacity of the bulk solution is strong enough, the pH reduction only takes place in dew droplets exposed to the CO_2 rich gas phase. To evaluate how much the CO_2 atmosphere also affected the bulk solution, the pH of the sample solution was measured before and after an experiment. To do this, the initial pH of a 45 µL of buffer solution was confirmed with a Thermo Scientific™ Orion™ 9826BN Micro pH Electrode (Thermo Fisher Scientific, USA). Subsequently the volume was split into three 15 µL aliquots and filled into an AWI with a triple chamber design. The AWI was then subjected to a heat gradient and a CO_2 flux identical to experimental conditions for RNA ligation. After four hours of runtime the volumes of the three chambers were extracted, combined and the pH measured again. These experiments were conducted on three different days, and since the influx of CO_2 was controlled manually, the intervals and lengths of the influx might have experienced slight variations. The median of the starting pH of the solution was 7.61 with an average pH drop of 0.57 ± 0.32 to a median of pH 7.07 after 4 h of incubation. From these control experiments we concluded that the buffer in the bulk solvent was sufficient to prevent significant global acidification.

3.2. Temperature simulations

The temperature gradient inside the chamber was modeled by finite-element simulations. A digital model was created using 3D CAD software (Autodesk Inventor 2021) and could be imported into the finite element software (COMSOL 5.4). The thermal conductivities for each element of the 3D model were taken from the COMSOL internal database or from the product data sheets of the materials used. The values for the thermal conductivities k [W/(m K)] at room temperature are: k_{H2O}= 0.62 W/(m K), k_{steel}= 44.5 W/(m K), $k_{aluminium}$= 237 W/(m K), $k_{sapphire}$= 35 W/(m K) and k_{FEP_foils}= 0.2 W/(m K). The heat flow through the outer surfaces of the full model was assumed to be thermally insulating, i.e. $Q_{extSurface}$ = 0. The temperature profile was then calculated following:

$$\nabla \cdot (k \nabla T) = -Q, \qquad (1)$$

where T [K] is the temperature, k [W/(m K)] the thermal conductivity and Q [W/m^3] the heat source. The temperature of the back side of the cold sapphire and the warm sapphire at the front side were fixed to the temperature measured at the back sapphire with a thermometer (Greisinger, GTH 1170) and with a heat camera (Seek Thermal, SQ-AAA), respectively. The measured temperatures were used as boundary conditions. The hot side of the sapphire was intentionally chosen to be the similar to the isothermal temperature. However, the cold temperature was optimized experimentally for good dew droplet dynamics. Good overall dynamics was found for the temperature combinations between 45 – 47 °C and 26 – 28 °C at the front and back sapphires,

respectively. Changes in temperature within this range did not appear to have a significant effect on the outcome of the reaction.

3.3. Supplementary Statistics and Reproducibility

The droplet dynamics shown in Supplementary Fig. 1c and 3 a–e are representative frames for all imaged AWI experiments (n>30). Each experiment consists of multiple hundred frames. Supplementary Fig. 5 shows one replicate of the ligation reaction with 5 mM $MgCl_2$ (n = 4), the template plus complementary control in the AWI system (n = 2) as well as isothermal conditions (n=2) and the no template control (n = 1). Supplementary Fig. 7 shows the independent replicates for the synthesis of A, B (n = 2), as well as C (n = 3). The synthesis reaction of C with a running time of 21h has been performed once (n = 1). Supplementary Fig. 8a was performed for two (n = 2) independent times for the reaction with template and once (n=1) for the reaction without template. In Supplementary Fig. 8 b) each concentration ratio was performed once. The gel of Supplementary Fig. c is representative for n = 2 independent experiments and is also shown in Figure 2b and c. All controls for unlabeled as well as FAM labeled HH-min synthesis shown in Supplementary Fig. 9 have been performed once (n = 1). For the kinetics in Supplementary Fig. 10 n = 2 independent experiments were performed for both, the AWI-system, as well as isothermal conditions at 50 mM $MgCl_2$. For the kinetics in Supplementary Fig. 11-12 of the HH-min synthesis by *sunY* in AWI systems, independent experiments were performed n = 3 for isothermal experiments with a $MgCl_2$ concentration of 25 mM and AWI experiments at 5 mM $MgCl_2$, n = 4 for isothermal with 50 mM $MgCl_2$ and n = 2 for 25 mM and 100 mM $MgCl_2$. For replicates in the AWI-system, separate AWI-systems were prepared with each AWI-system corresponding to one time point and each reaction ran in a separate chamber (Supplementary Figs. 10-12). For Supplementary Fig. 13a, the gel stands for n = 4 independent experiments for both the 2h and 4h time point. For Supplementary Fig. 13b, the 2h and 4h lane (+*sunY*) of the AWI-system are representative gels of n = 10 and n = 4 independent experiments, respectively. Here, the -*sunY* controls were each performed once. The isothermal control was performed in a total number of 4 independent experiments for both concentration ratios (with each n = 2). In Supplementary Fig. 14a each experiment was performed once. In Supplementary Fig. 14c and d, isothermal controls were performed once each. The AWI experiments were performed with n = 2 independent experiments.

4. Supplementary Notes

4.1. Supplementary Note 1

We evaluated the equilibrium base pairing for the sequence design of all systems using the NUPACK analysis tool[5]. The algorithm is based on free energy calculations including nearest-neighbor empirical data of Serra and Turner (1995). However, the parameters are set for salt concentrations of 1 mM Na^+, thus, generally underestimating the binding stability of our system as the presence of magnesium ions leads to a higher duplex stability. The temperature was fixed to 25 °C and the maximum complex size was set to 4. NUPACK predicts minimal free energy (MFE) structures and base-pairing probabilities of RNA sequences for a given set of input parameters (salt concentration, temperature, maximum complex size, sequences and strand concentrations). The prediction additionally outputs the equilibrium concentrations of the MFE complexes and their associated free energy of folding. For the model ligation system, the experimental concentrations of 5 µM for template and 20 µM for each of the three ligation substrates were given as input parameters. The equilibrium concentrations of the different predicted MFEs where the cutoff for the fraction of maximum concentration was set to 0.005. At 25 °C, 96% of the templates are bound to the three substrates. The mean free energy of this structure is -53.07 kcal/mol. Another complex, T-M1-M2-M2, appears in the histogram, if the cutoff of the fraction of maximum concentration was lowered to 0.001. With an equilibrium concentration of 0.024 µM this complex is negligible but could lead to some unwanted side product formation. For the ABC system, several scenarios were simulated. Generally, the input concentrations of 2.5 µM for the template and 10 µM for each of the three ligation substrates were chosen again to match the experimental setup. Additionally, the sequence of the *sunY* ribozyme was included in the simulation with an input concentration of 2.5 µM to estimate the inhibitory effect of the complementary templates on *sunY*. Initially, the equilibrium concentrations for each of the *sunY* ligation substrates were calculated individually, i.e. (tA123, A1, A2, A3, *sunY*), (tB, B1, B2, B3, *sunY*), (tC123, C1, C2, C3, *sunY*). The A123 fragment of the *sunY* as reported in[6] has slight sequence variations compared to the full-length version as used here and reported in[2]. This is reflected in the outcome of the NUPACK simulation. Again, the cutoff for the fraction of maximum concentration was set to 0.005. At 25 °C, almost all templates are bound to all three ligation substrates. The mean free energy of this structure is -80.27 kcal/mol. In contrast, *sunY* is predicted to be mostly present in its active form and barely inhibited. However, simulating the mean free energy structures for tA123 and *sunY* as well as tA123, tB123, tC123 and *sunY* results in structures with a free energy of -114.47 kcal/mol and -254.74 kcal/mol, respectively. This suggests that at least some of the *sunY* is inhibited by tA123. For fragment B123 and C123, the inhibitory effect according to the mean free energy structures is significantly stronger, as most of the *sunY* is present in some bound form to either tB123 or tC123. For both simulations, the temperature was set to 25 °C and cutoff for the fraction of maximum concentration was set to 0.005. For the hammerhead system, an input concentration of 5 µM for the template and 10 µM for each of the three ligation substrates were chosen to match the experiment. Again, the cutoff for the fraction of maximum concentration was set to 0.005. At 25 °C, 100% of the templates are bound to all three ligation substrates. The mean free energy of this structure is -82.89 kcal/mol.

For additional information on the thermal stability of the model ligation system, melting temperatures were also experimentally measured using a C1000™ Thermal Cycler with a CFX96™ detection system from Bio-Rad Laboratories (BioRad CFX Manager 3.1).

Melting curves were derived from the change of fluorescence of 1x EvaGreen Plus dye (stock at 20X in Water, Biotium). The concentrations of template and *Full complement* were kept at 5 µM, the concentration of substrates was kept at 20 µM. Three different compositions were measured, *Template + Substrates*, *Template + Full complement* and *Template + Substrates + Full complement*. In each sample the buffer concentrations were 30 mM Tris·HCl pH 7.5, 100 mM KCl and 1 mM $MgCl_2$. Higher magnesium concentrations did not yield a sufficient plateau to estimate the melting temperature. To limit the exposure to high temperatures, the temperature range was 4 °C-70 °C with a cooling and heating rate of 1 °C / 30 sec. For template plus its full complement, no second plateau is visible in the fluorescence signal. Therefore, the melting temperature cannot directly be extracted and is likely above 70 °C. For *Template + Substrates*, the fluorescence signal shows a sigmoidal behavior with two plateaus. The respective fraction bound after the baseline correction (red dots) and was fitted (solid red line) using the following equation:

$$f(T) = 1 - \frac{1}{1 + \exp\left(\frac{T_m - T}{r}\right)}, \qquad (2)$$

where the fitting parameters are the melting temperature (T_m) and rate. For the case of *Template + Substrate + Full complement* two transitions are expected, the first one corresponding to the unbinding template and substrates, the second to unbinding template and full complement. It was possible to adjust the baselines such that a fraction bound could be extracted for the first part and was fitted as described above. The corresponding melting temperatures are T_m = 49.6 °C and T_m = 46.8°C for *Template + Substrates* and *Template + Substrates + Full complement*, respectively.

4.2. Supplementary Note 2

The sequences and the binding sites of the ligation substrates for the ligation of A123 and tA123 are shown below. As the ligation site between tA1 and tA2 has a minimum of 7 nt overlap with A2, this ligation step of the complementary site can proceed without strand separation and is present to some extent in all reactions (AWI and isothermal).

<u>Complementary ligation substrates for sense and antisense strand of *sunY* fragment A:</u>

tA1 | tA2 | tA3

```
   5’FAM-ugauuuagc gacggau|ugucucagugag ggu|uucccguuuuag-3
3’-augacgacuaaaucg|uugccua acagagucacuc|uca aagggcaaaauccgucuaaaag5’ - Cy5
```

A3 | A2 | A1

The sequences and the binding sites of the ligation substrates for the ligation of C123 and tC123are shown below. Compared to ligation substrates for A, the ligation sites have shorter overhangs.

<u>Complementary ligation substrates for sense and antisense strand of *sunY* fragment C:</u>

tC1 | tC2 | tC3

```
5’FAM-cugcauguc gcca|ugcaguucag gcu|auaucuucaa-3’
3'cuaggacguacag|uggu acgucaaguc|uga uauagaaguugagaa-Cy5-5’
```

C3 | C2 | C1

4.3. Supplementary Note 3

The temperature gradient across the compartment leads to evaporation and re-condensation of water at the hot and cold side of the chamber, respectively. The resulting dew droplets at the cold side can grow and coalesce. This dynamic depends on the pressure and type of gas, the temperature gradient, and the thickness of the chamber and can be monitored by microscopy, either in bright field or in fluorescence mode. If the droplets grow sufficiently large, they can either precipitate back into the bulk solution (precipitation) or rearrange due to surface tension, such that they touch both sides of the chamber. Dew droplets that adhere to both windows either precipitate back into the bulk solution or dry. Monitoring the fluorescence of Cy5 labeled RNA shows that temperature gradients also trigger the accumulation of RNA at the water-gas interface. Upon movement of the interface this leads to some dried or semi-dried material at the interface. This material can be re-dissolved and transported back into solution by dew droplets that touch the warm side. The droplet dynamics are a crucial parameter for the respective experiment as they are responsible for the strand-separating conditions. The top temperature of the gradient for all experiments presented here was chosen such that it matches the isothermal control. The best cold temperature was screened by monitoring the droplet dynamics of buffer and adapted according to the best droplet dynamics.

Supplementary References

1. Matreux, T. *et al.* Heat flows in rock cracks naturally optimize salt compositions for ribozymes. *Nat. Chem.* **13**, 1038–1045 (2021).
2. Doudna, J. A., Usman, N. & Szostak, J. W. Ribozyme-Catalyzed Primer Extension by Trinucleotides: A Model for the RNA-Catalyzed Replication of RNA. *Biochemistry* **32**, 2111–2115 (1993).
3. Drobot, B. *et al.* Compartmentalised RNA catalysis in membrane-free coacervate protocells. *Nat. Commun.* **9**, 3643 (2018).
4. Ianeselli, A. *et al.* Water cycles in a Hadean CO2 atmosphere drive the evolution of long DNA. *Nat. Phys.* **18**, 579-585 (2022) doi:10.1038/s41567-022-01516-z.
5. Zadeh, J. N. *et al.* NUPACK: Analysis and design of nucleic acid systems. *J. Comput. Chem.* **32**, 170–173 (2011).
6. Doudna, J., Couture, S. & Szostak, J. A multisubunit ribozyme that is a catalyst of and template for complementary strand RNA synthesis. *Science* **251**, 1605–1608 (1991).

Appendix

Supplementary Fig. 5a

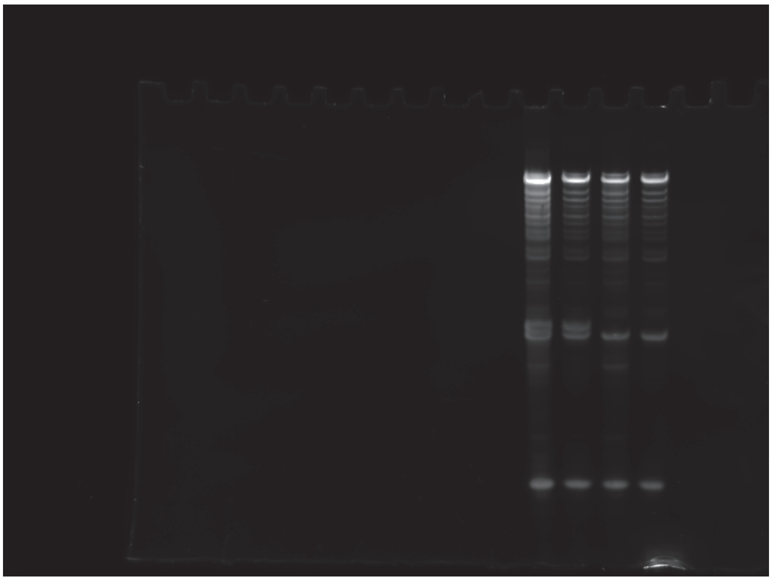

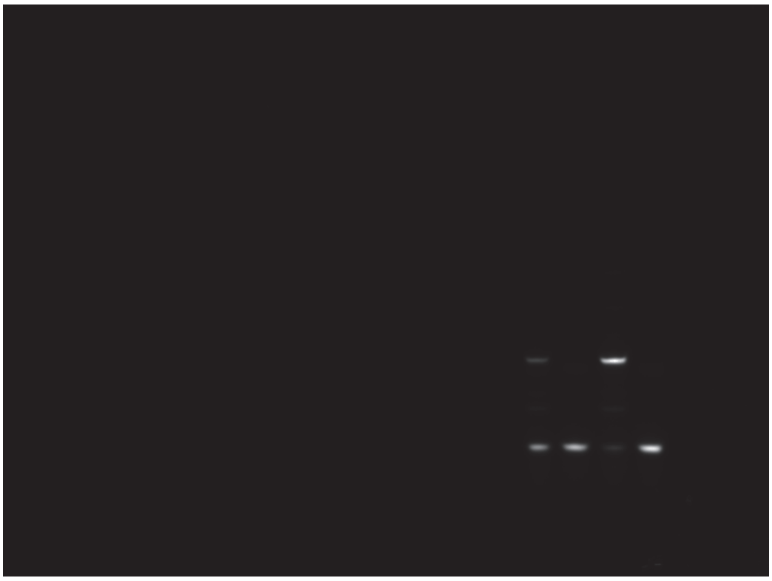

Supplementary Fig. 5b

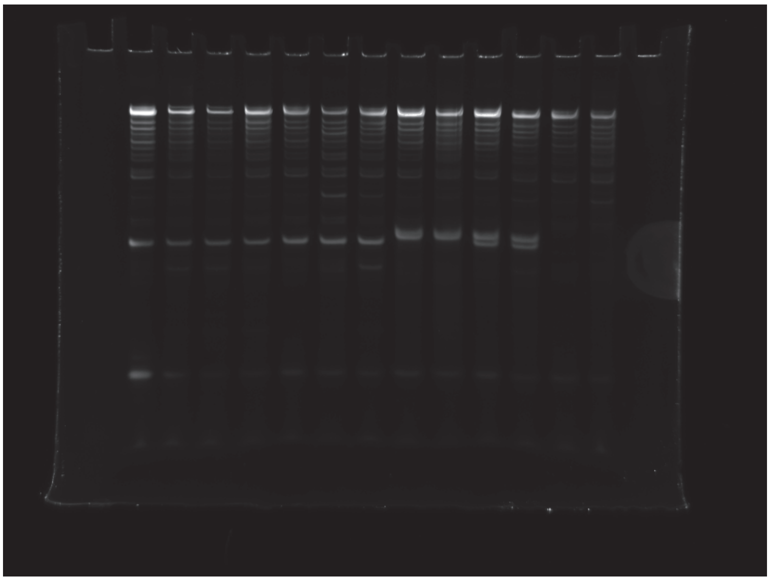

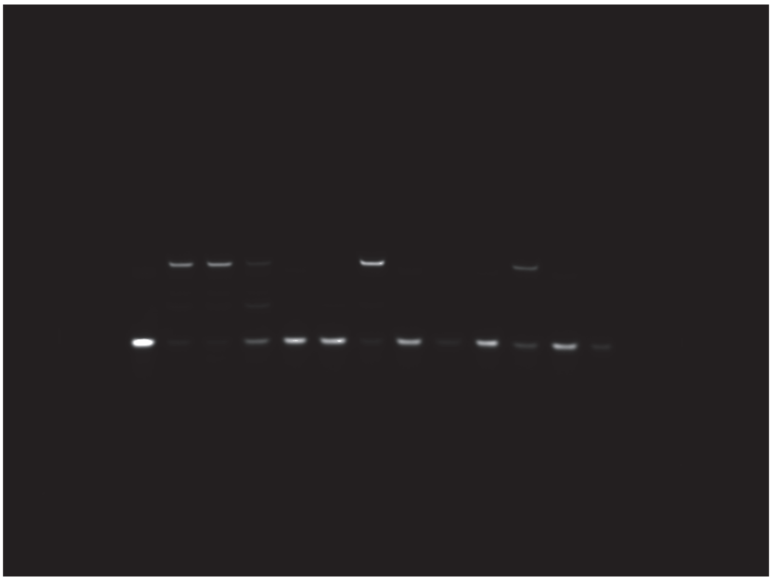

Supplementary Fig. 7b, c

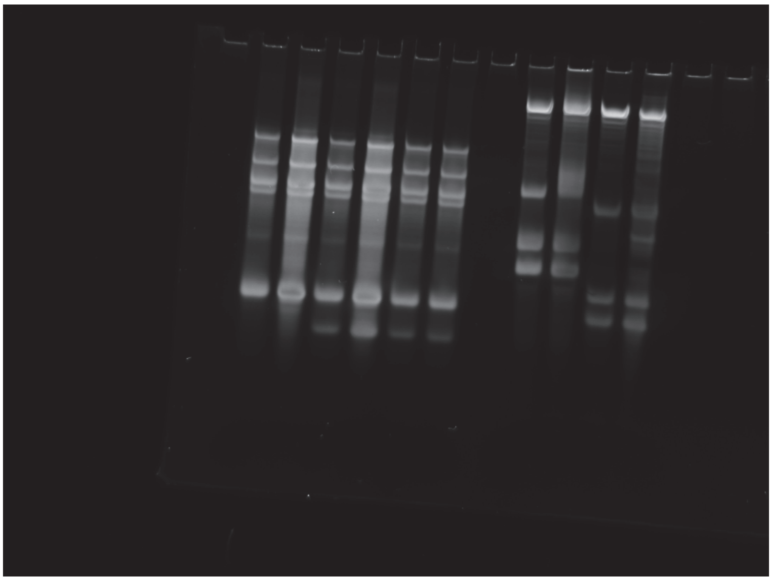

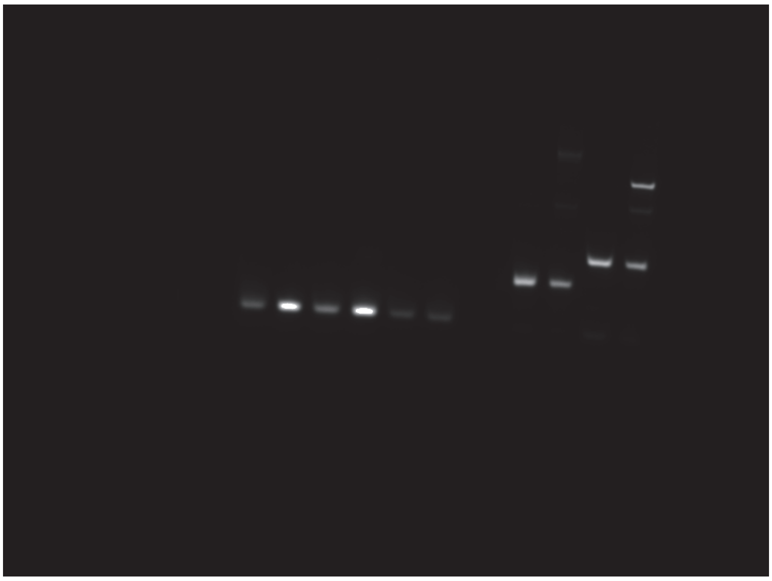

Supplementary Fig. 7f

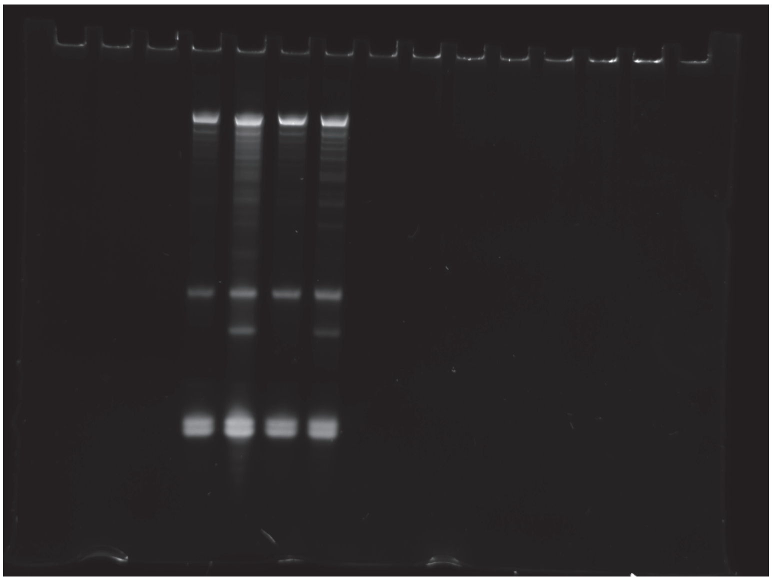

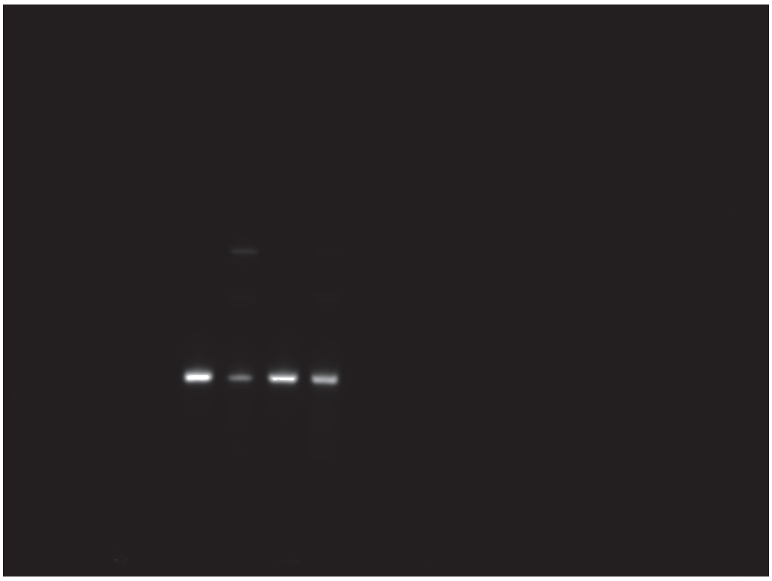

Supplementary Fig. 7 a, d

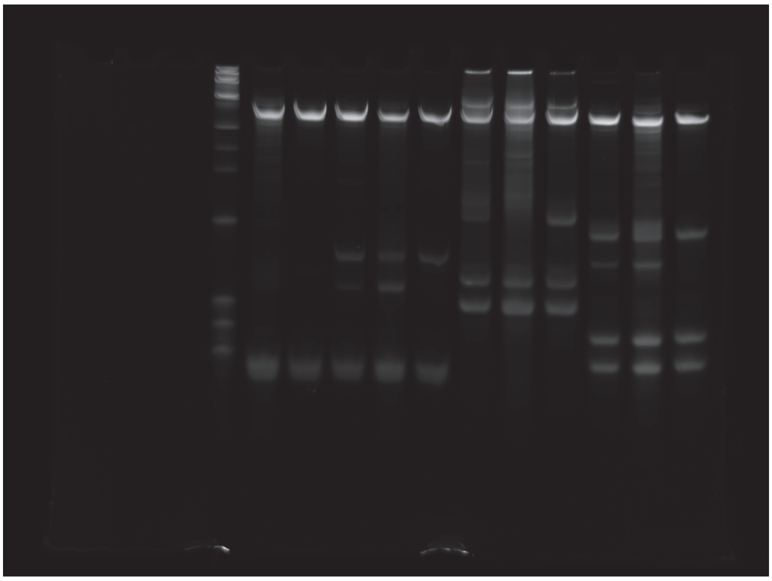

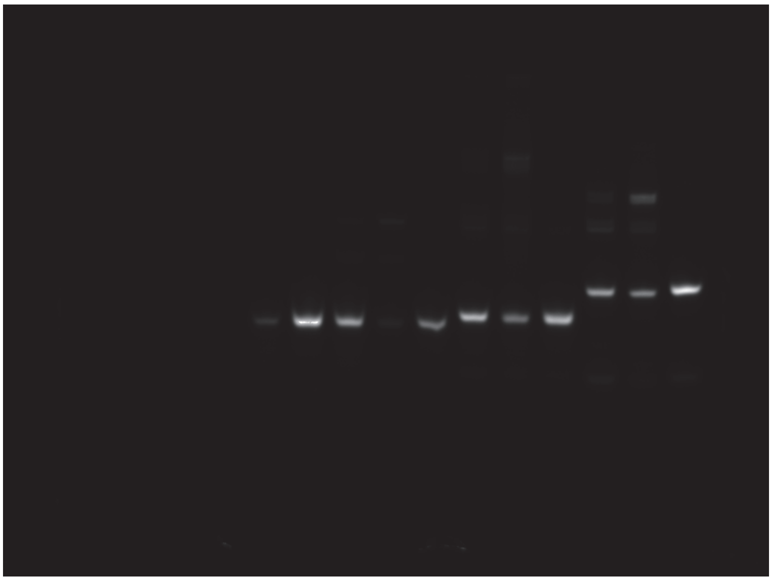

Supplementary Fig. 7e

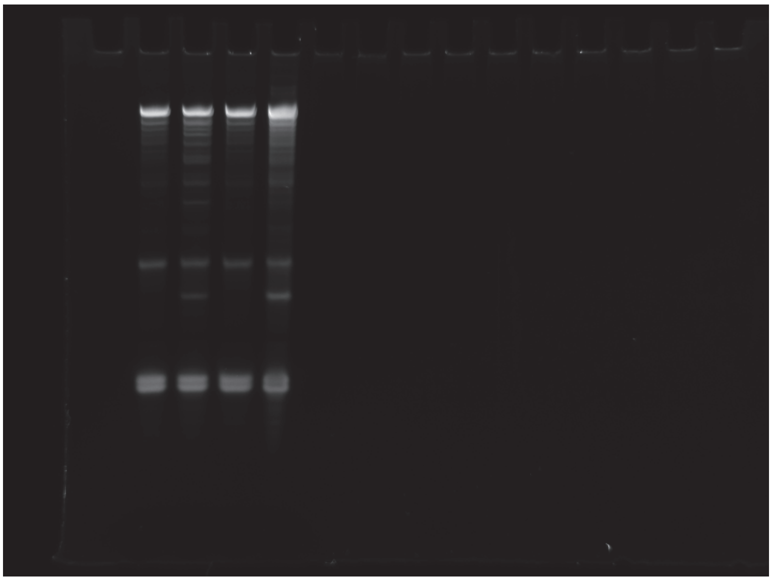

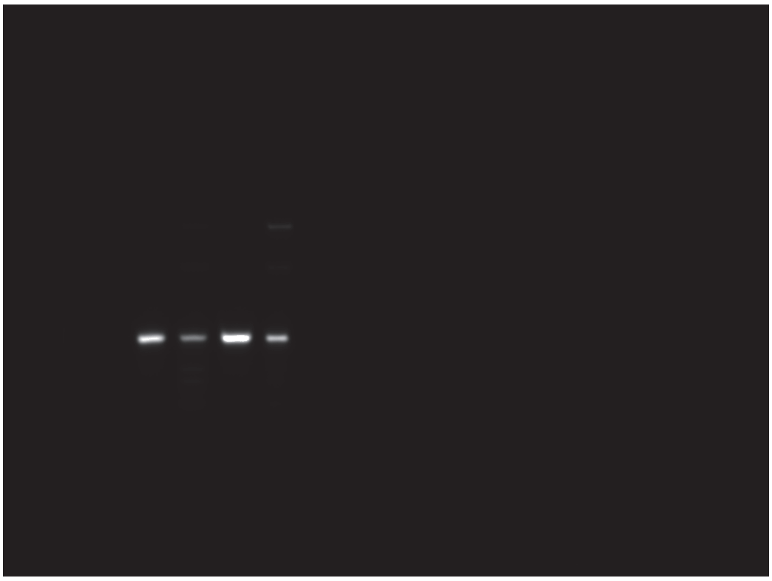

Supplementary Fig. 7g

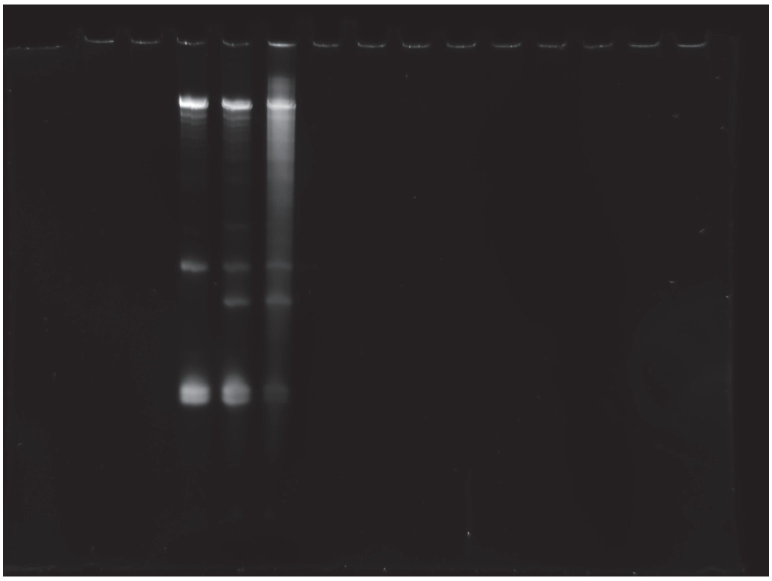

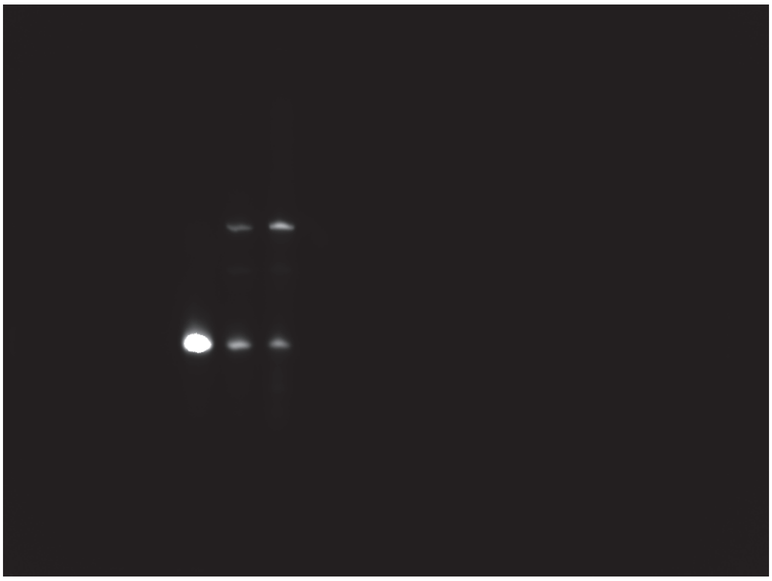

Supplementary Fig. 8a

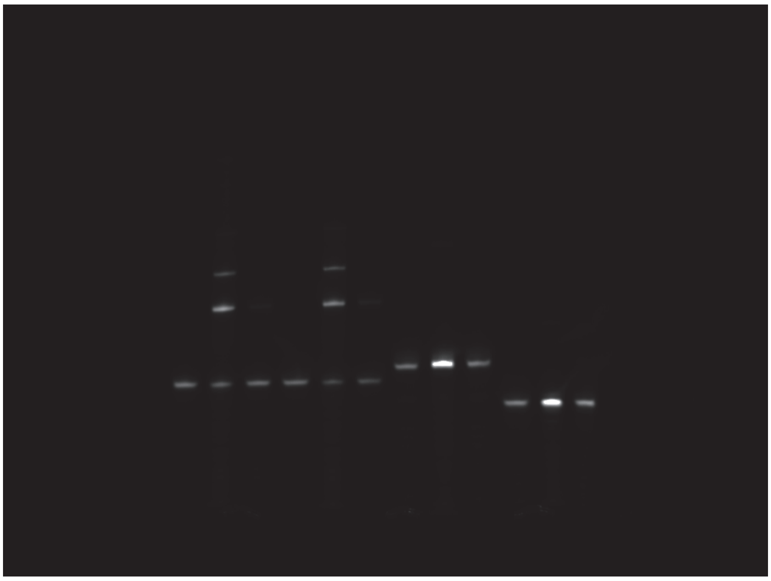

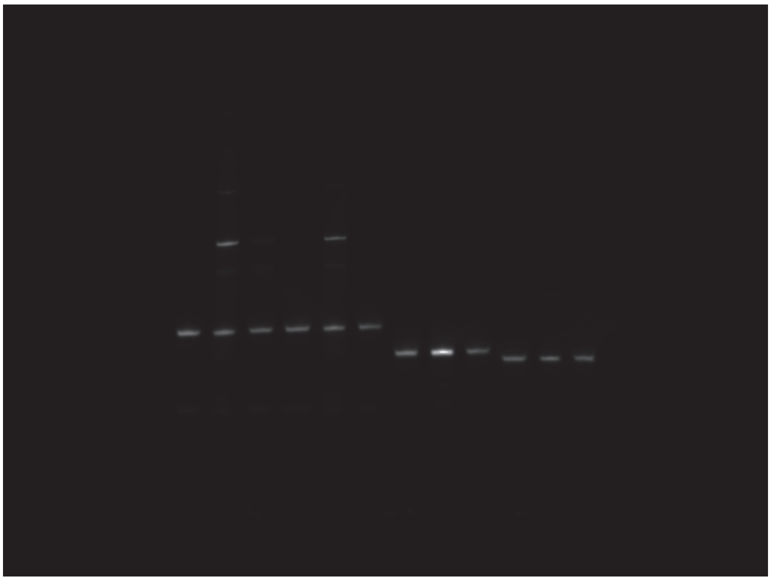

Supplementary Fig. 8b

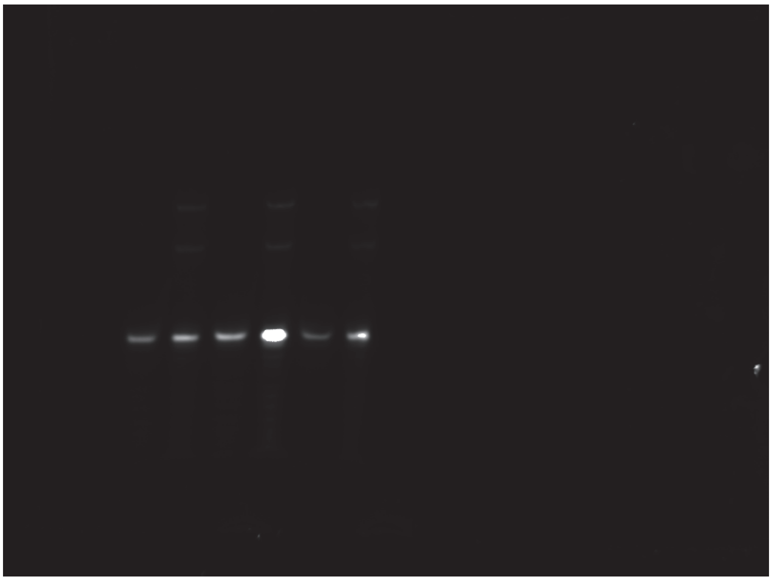

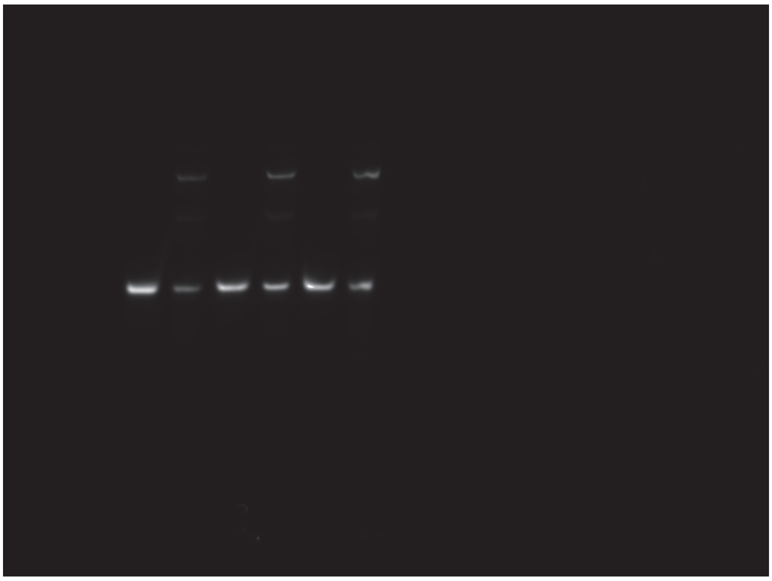

Supplementary Fig. 8c

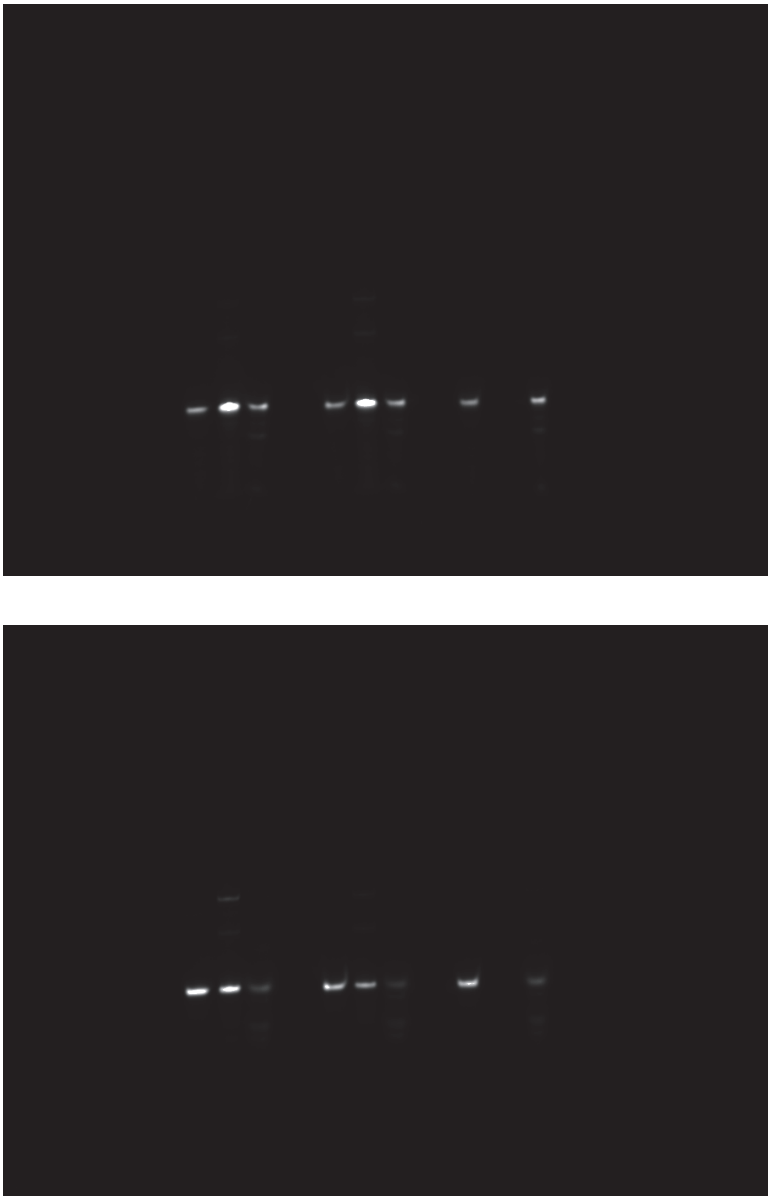

Supplementary Fig. 8d

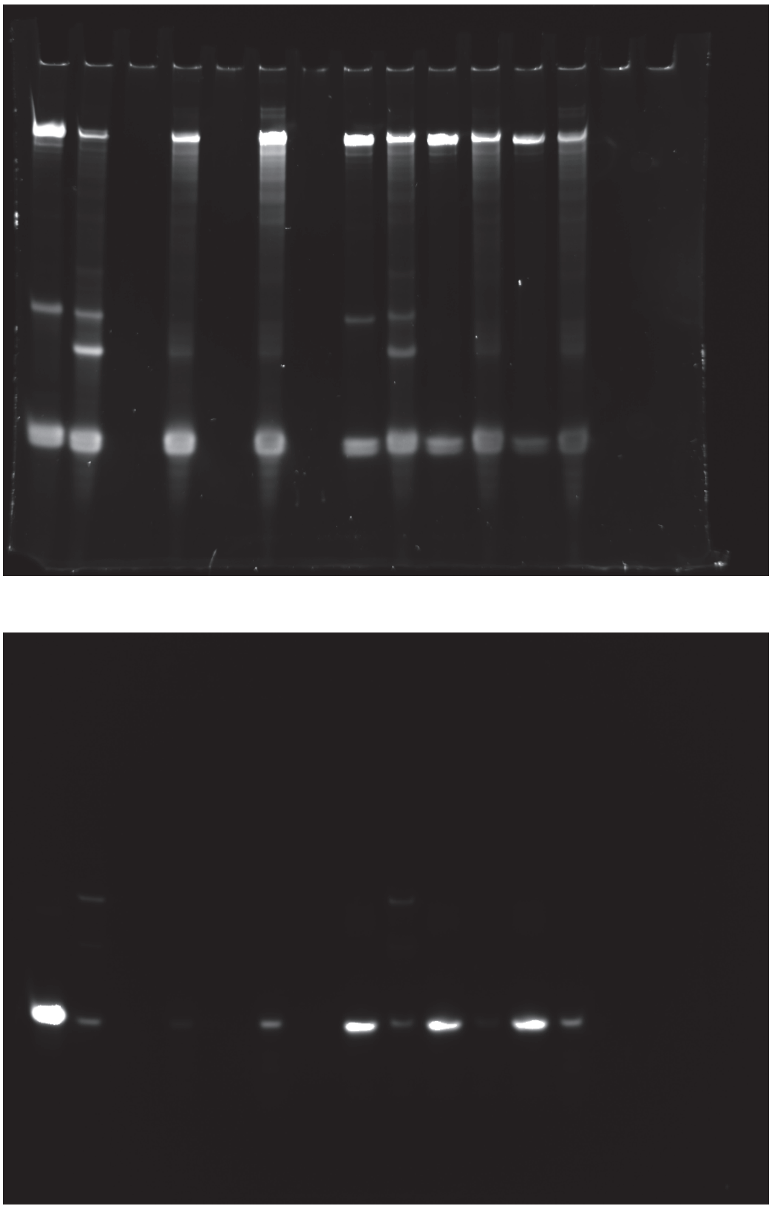

Supplementary Fig. 9a

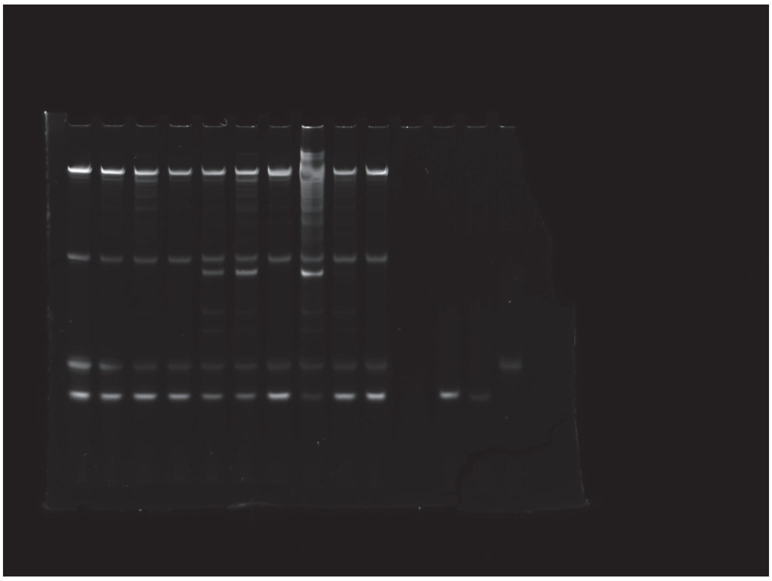

Supplementary Fig. 9b

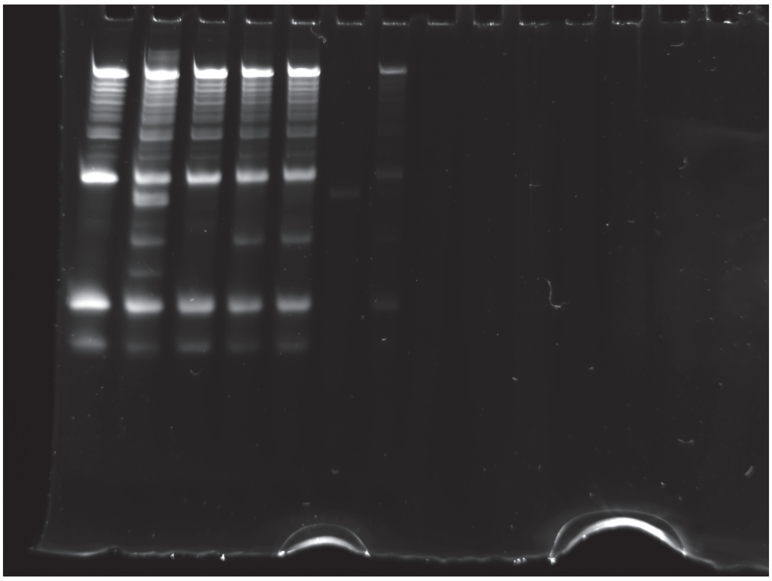

Supplementary Fig. 9 c

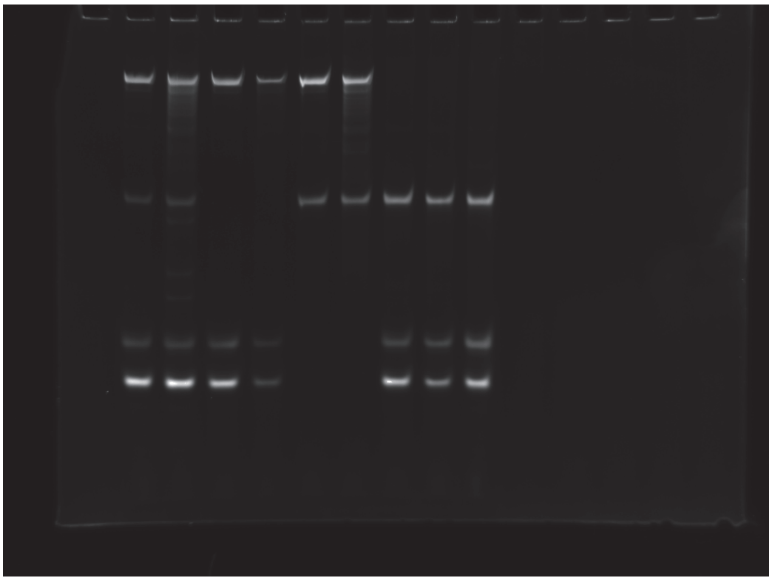

Supplementary Fig. 10c

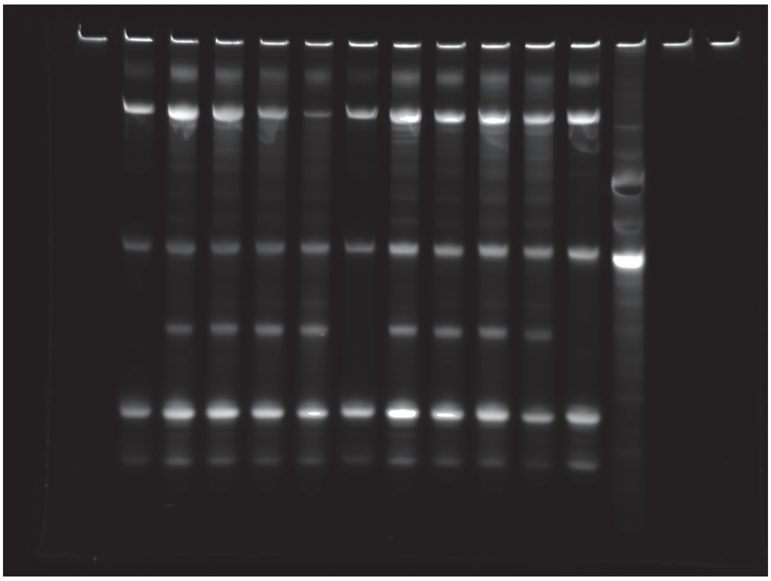

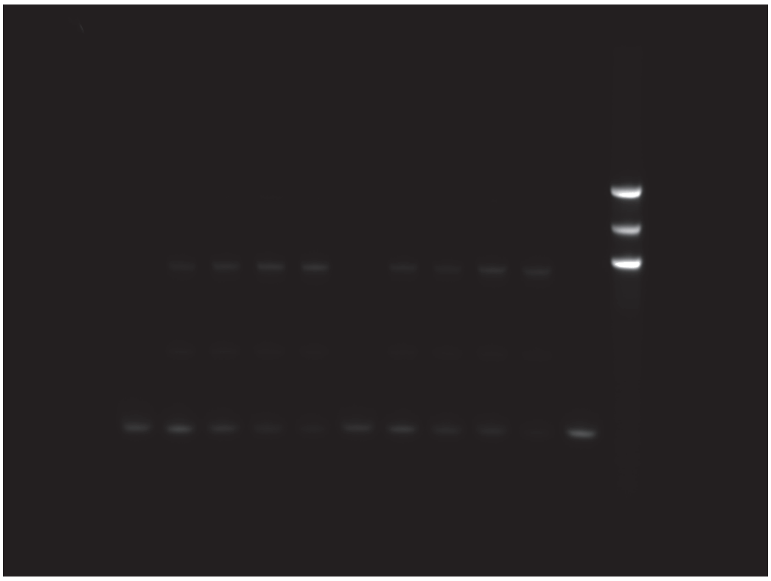

Supplementary Fig. 10d

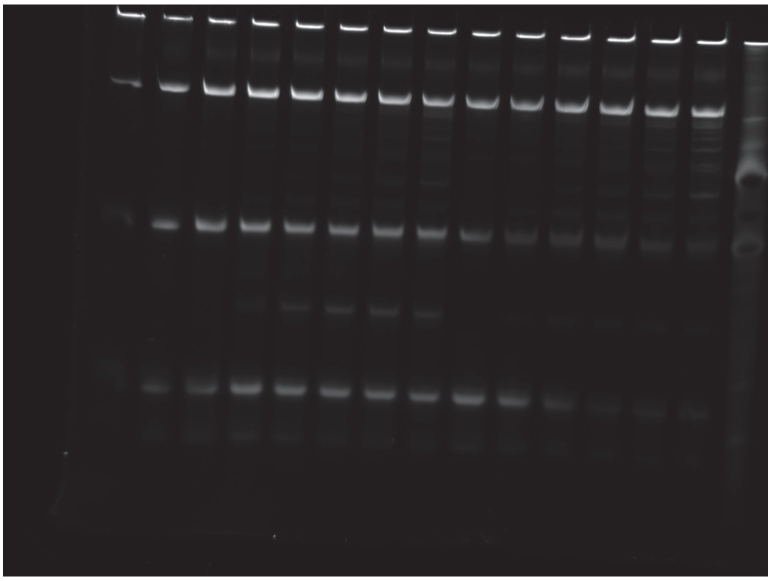

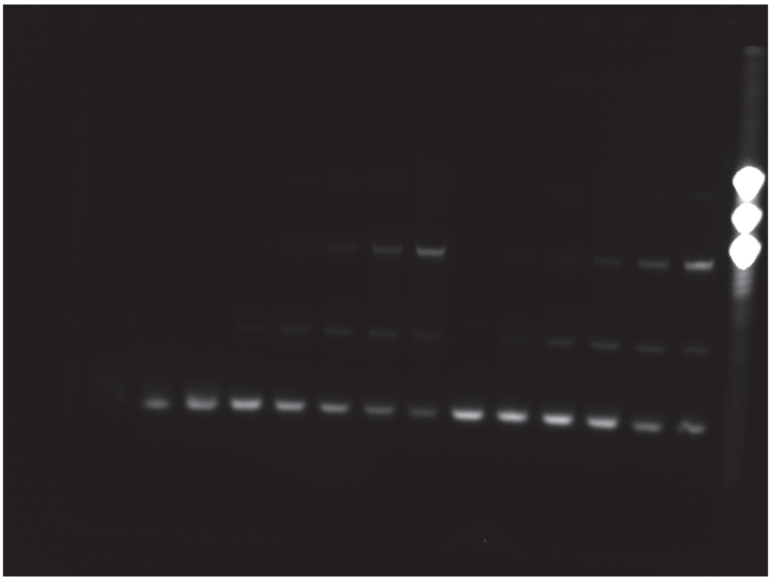

Supplementary Fig. 11a

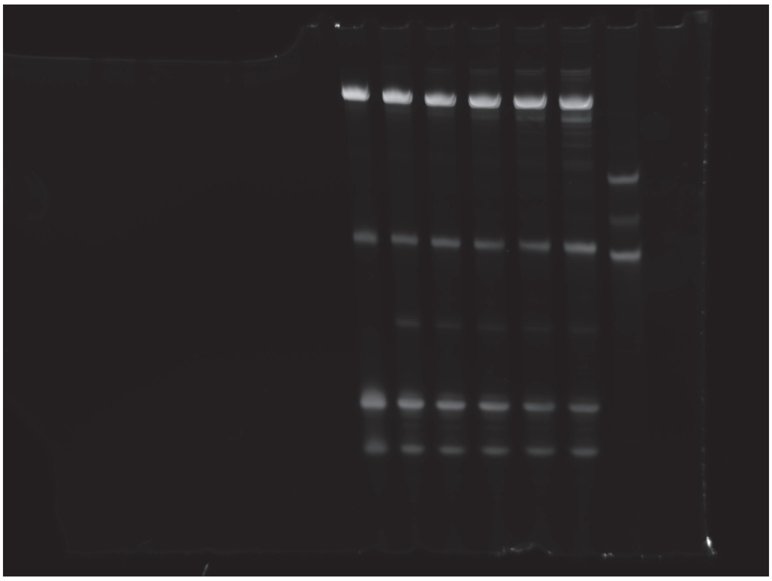

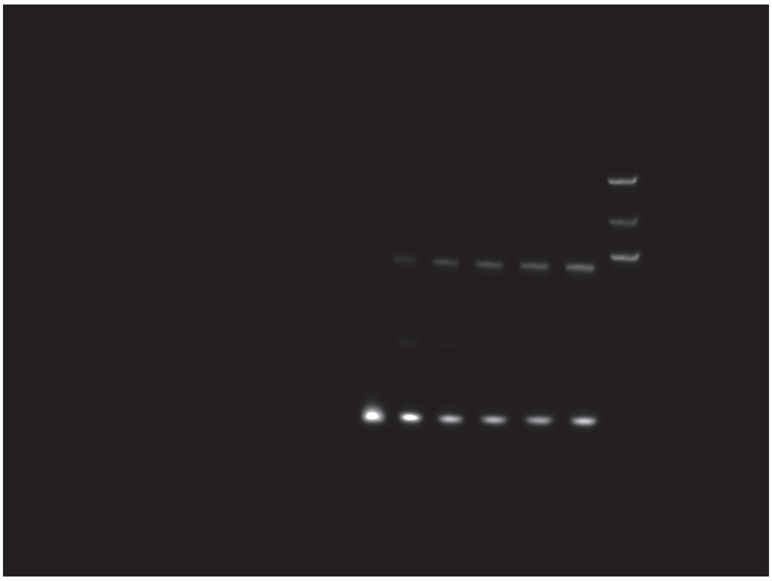

Supplementary Fig. 11b

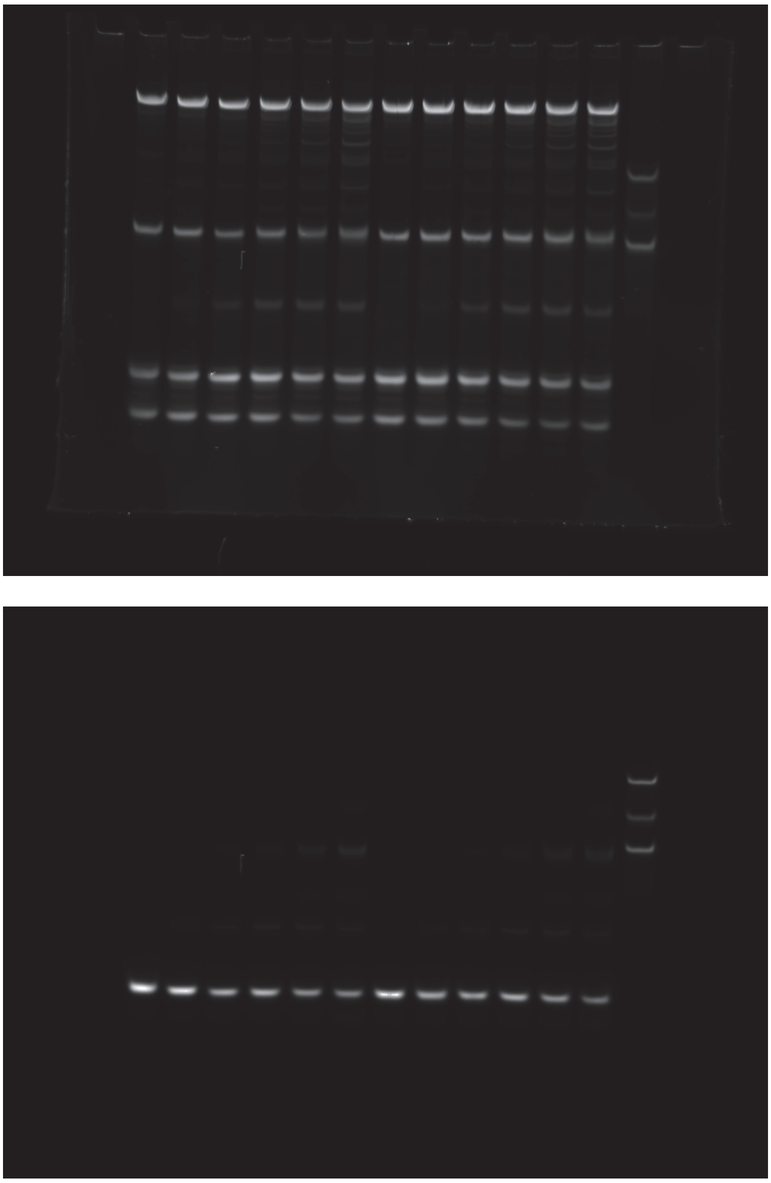

Supplementary Fig. 11c

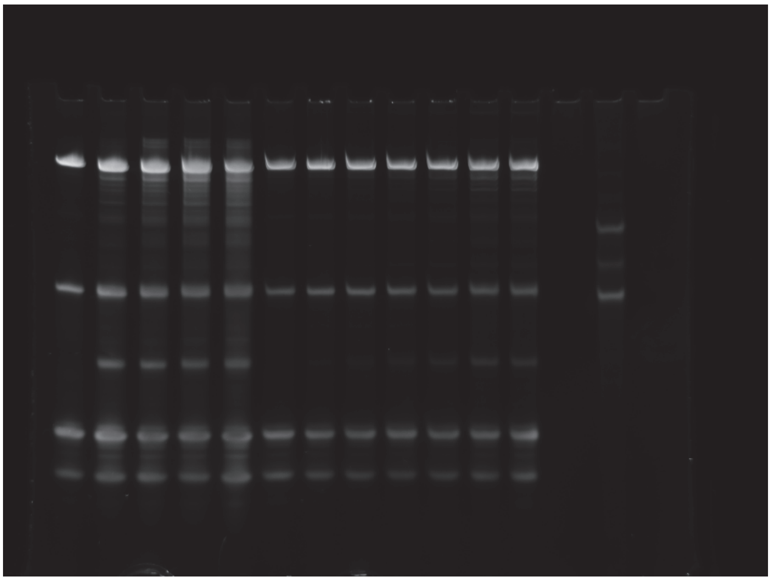

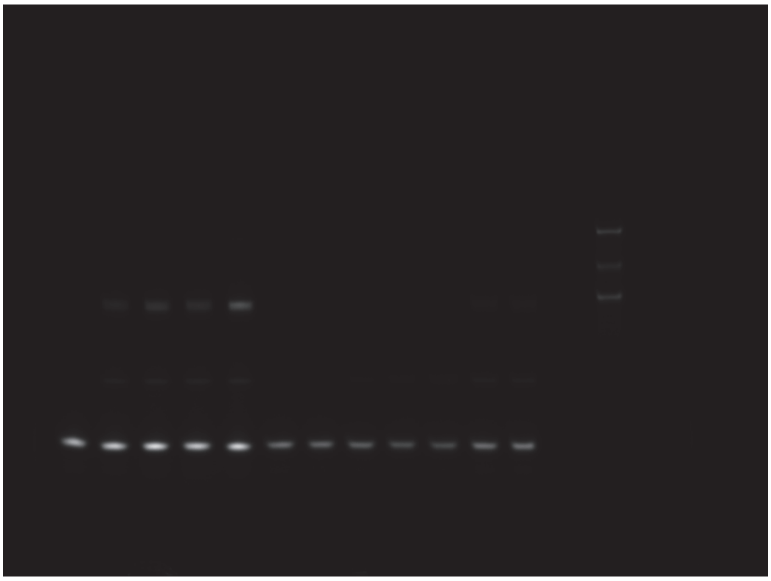

Supplementary Fig. 11d

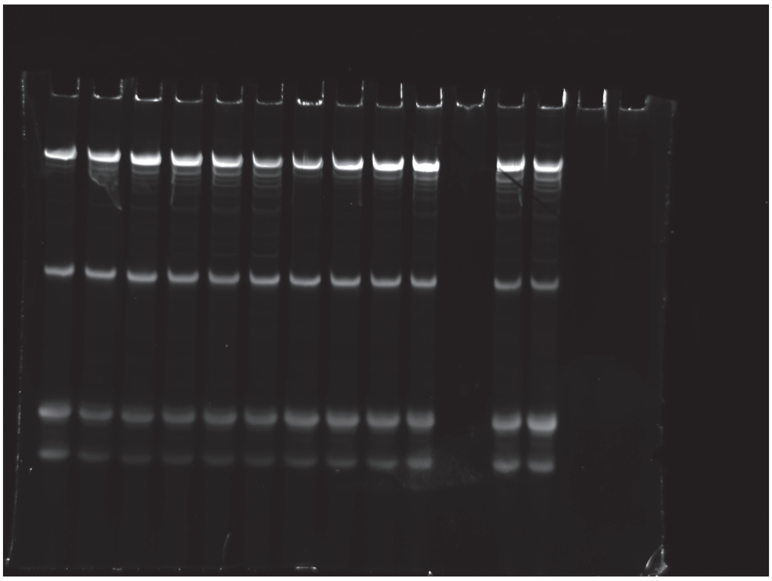

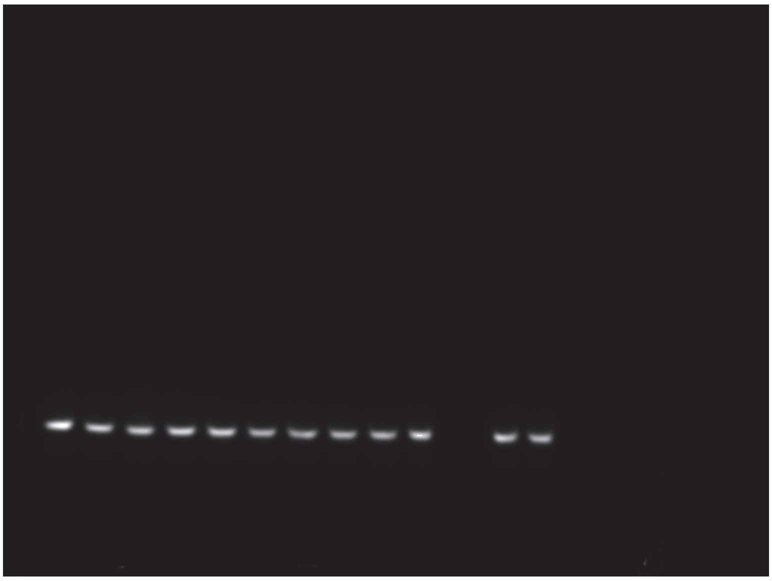

Supplementary Fig. 11e

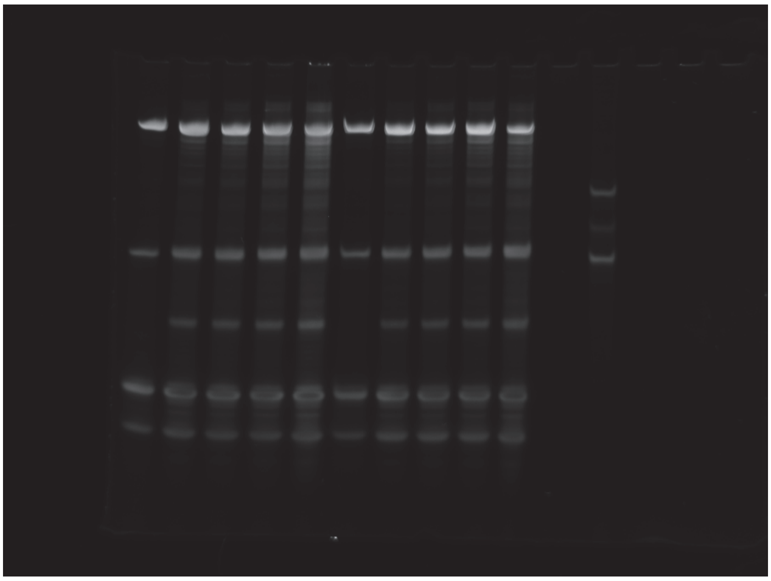

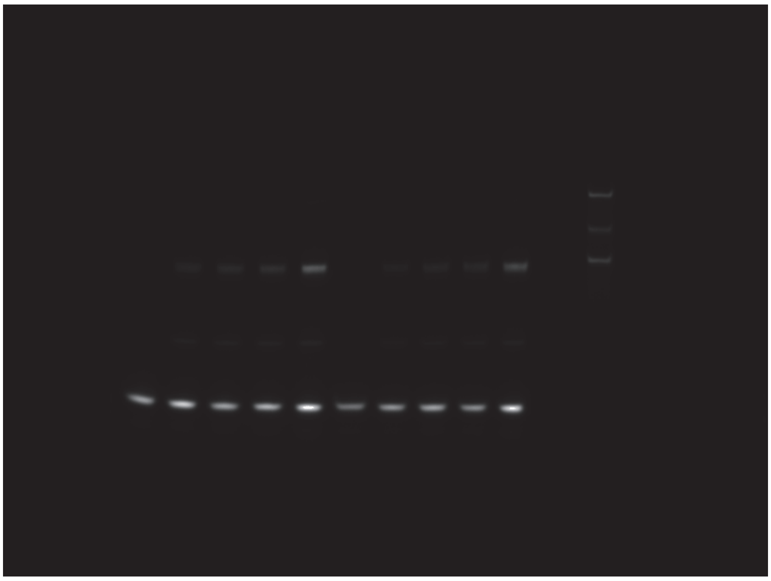

Supplementary Fig. 13a, b

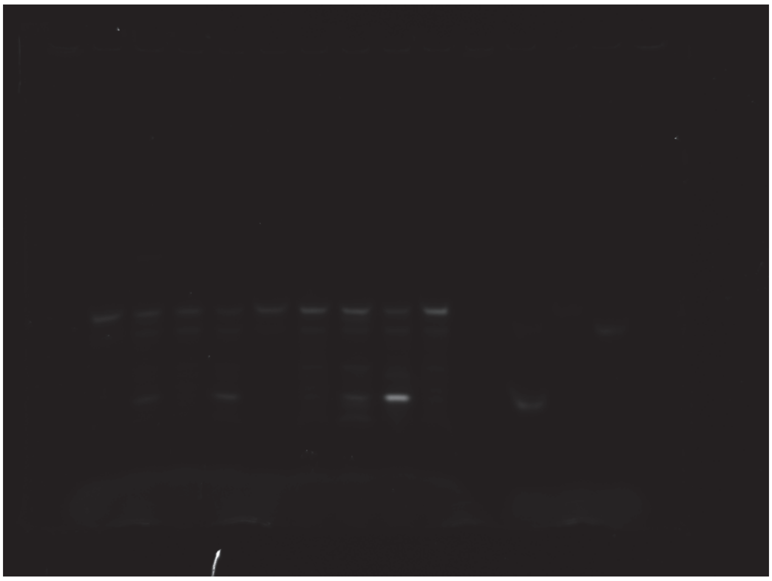

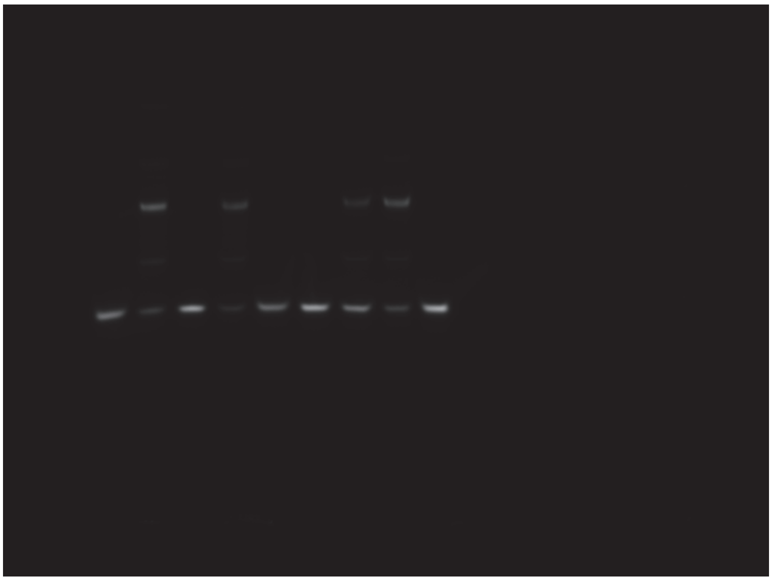

Supplementary Fig. 13d

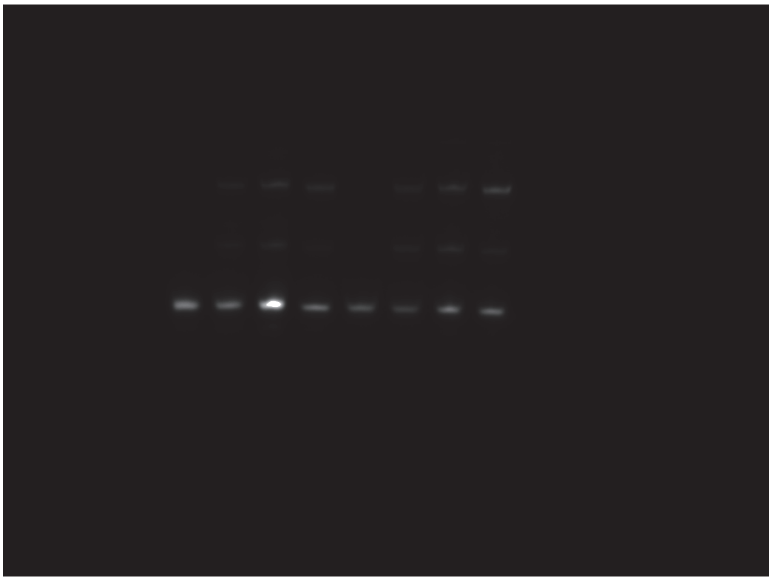

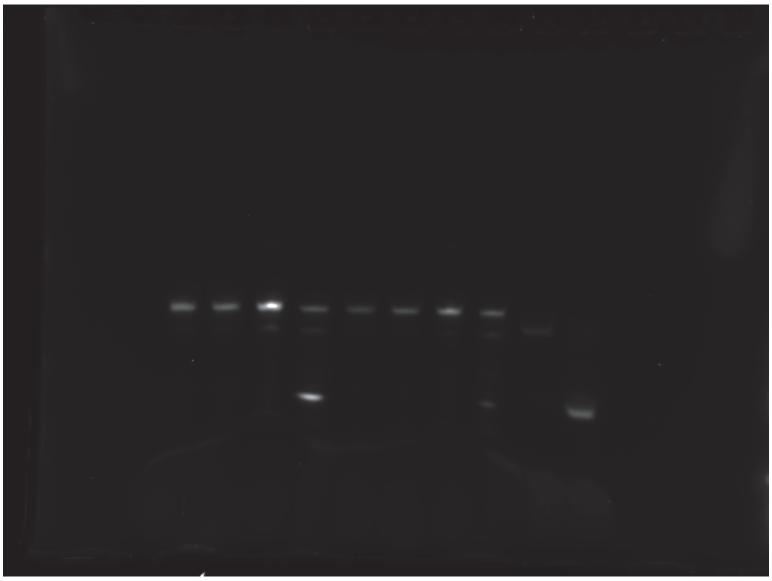

Supplementary Fig. 14a

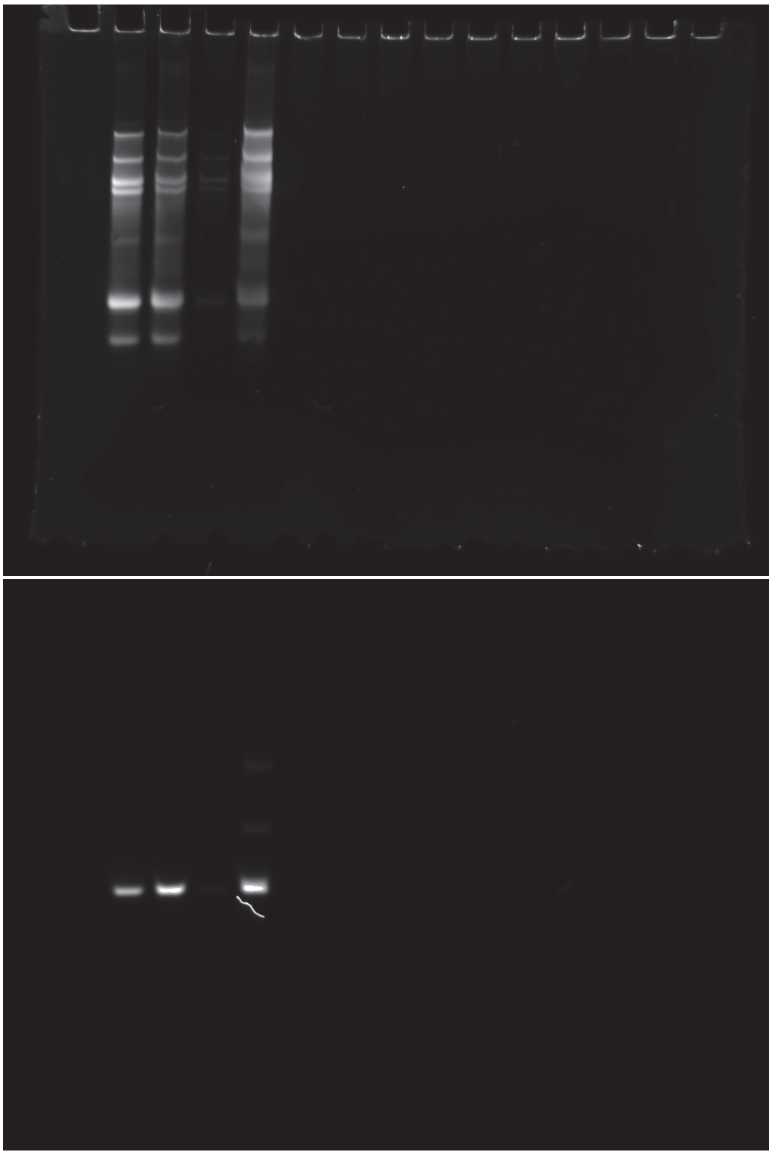

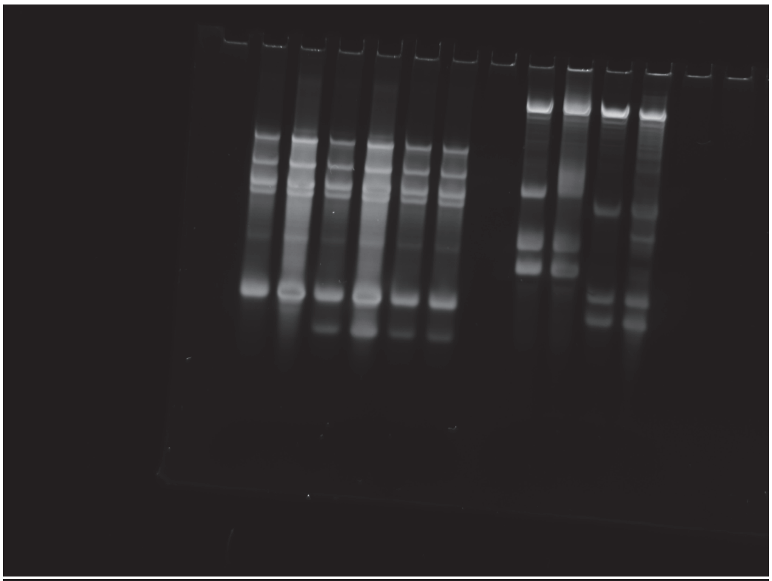

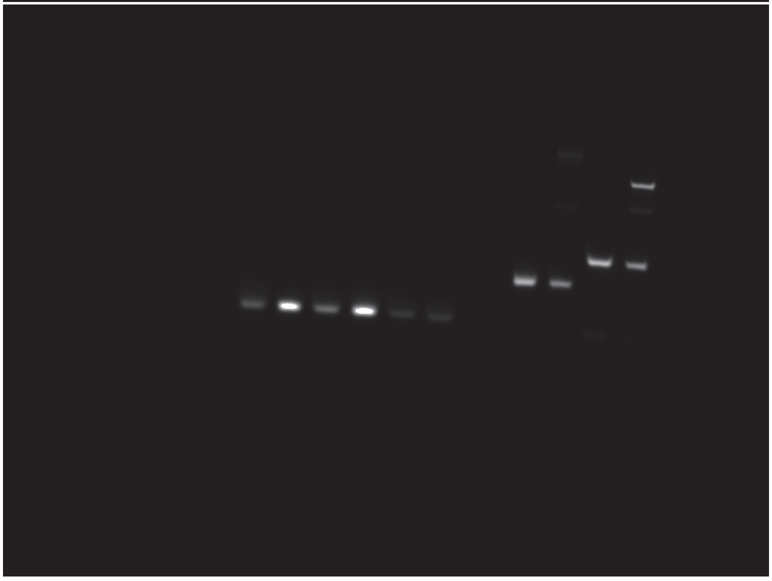

Supplementary Fig. 14b, c

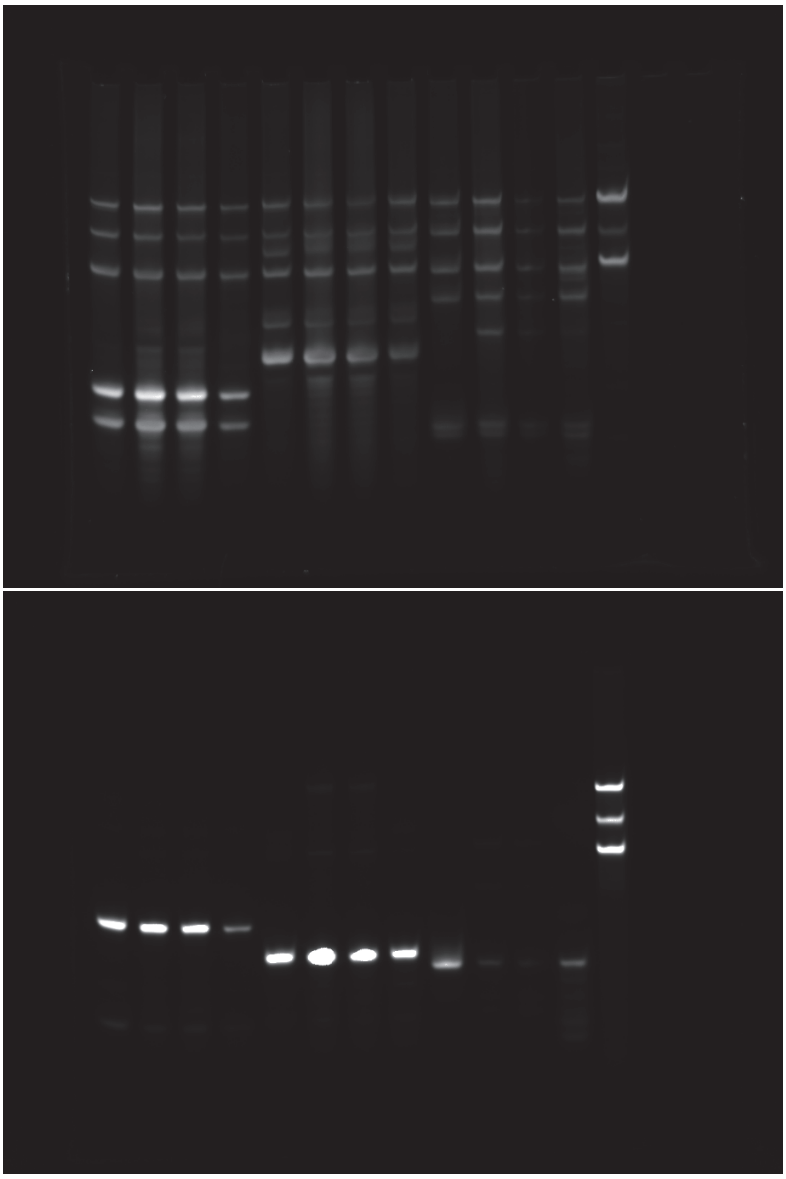

Supplementary Fig. 14d

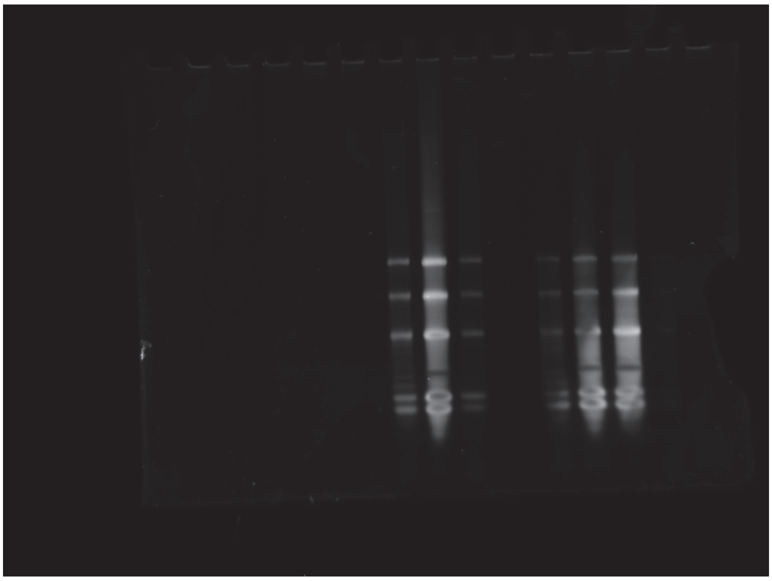

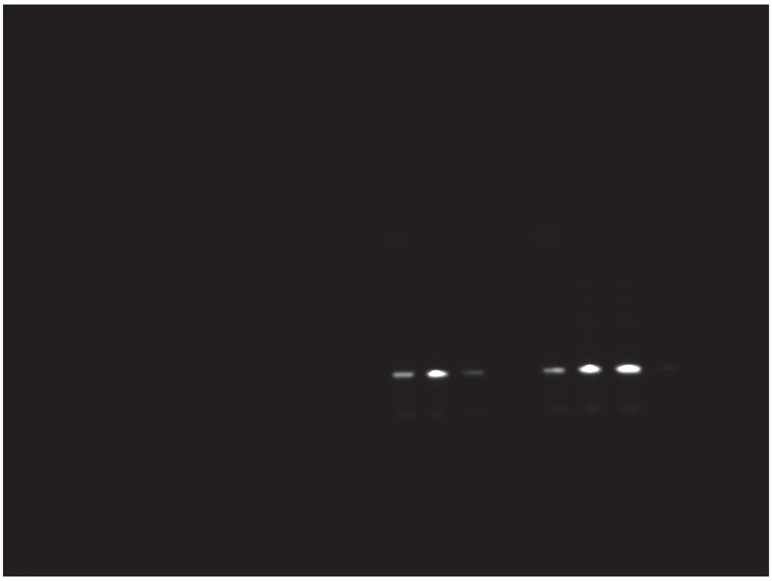

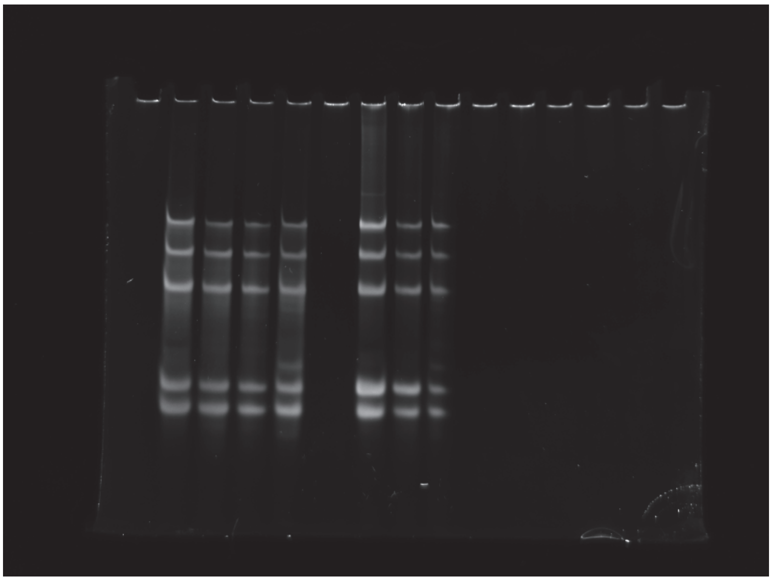

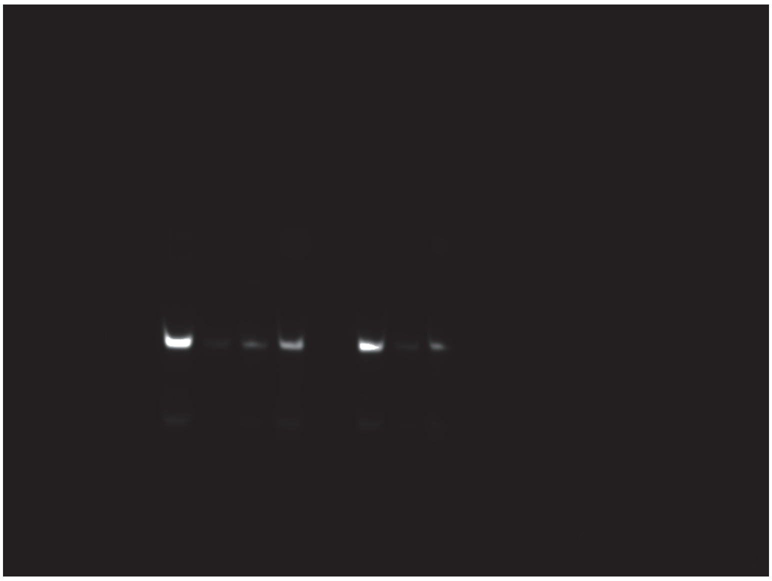

Supplement: Supplementary file 1 — Supplementary Information [file 41467_2023_37206_MOESM1_ESM.pdf]
